# Unraveling Cathepsin S regulation in interleukin-7-mediated anti-tumor immunity reveals its targeting potential against oral cancer

**DOI:** 10.1186/s12929-025-01154-6

**Published:** 2025-07-24

**Authors:** Yung-Chieh Chang, Szu-Jung Chen, Shang-Hung Chen, Sheng-Yen Hsiao, Li-Hsien Chen, Chung-Hsing Chen, Chan-Chuan Liu, Ya-Wen Chen, Ko-Jiunn Liu, Shang-Yin Wu, Jui-Mei Chu, Li-Ying Qiu, Wei-Fan Chiang, Hsing-Pang Hsieh, Wen-Yun Hsueh, Jenn-Ren Hsiao, Meng-Ru Shen, Jang-Yang Chang, Kwang-Yu Chang

**Affiliations:** 1 TMU Research Center of Cancer Translational Medicine; Taipei Cancer Center, College of Medicine, Taipei Medical University Hospital, Taipei Medical University, No. 250, Wuxing Street, Taipei, 11031 Taiwan; 2 National Institute of Cancer Research, National Health Research Institutes, 367 Sheng-Li Road, Tainan, 70456 Taiwan; 3 Institute of Biotechnology and Pharmaceutical Research, National Health Research Institutes, Miaoli, Taiwan; 4 Department of Oncology, Neurosurgery, Albert Einstein College of Medicine, New York, NY USA; 5 Department of Oncology, College of Medicine, National Cheng Kung University Hospital, National Cheng Kung University, Tainan, Taiwan; 6 Division of Hematology-Oncology, Department of Internal Medicine, Chi Mei Medical Center, Liouying, Tainan, Taiwan; 7 Department of Pharmacology, College of Medicine, National Cheng Kung University, Tainan, Taiwan; 8 Department of Oral and Maxillofacial Surgery, Chi Mei Medical Center, Liouying, Tainan, Taiwan; 9 School of Dentistry, National Yang Ming University, Taipei, Taiwan; 10 Department of Otolaryngology, College of Medicine, National Cheng Kung University Hospital, National Cheng Kung University, Tainan, Taiwan; 11 Institue of Clinical Medicine, College of Medicine, National Cheng Kung University, Tainan, Taiwan; 12 Department of Obstetrics and Gynecology, College of Medicine, National Cheng Kung University Hospital, National Cheng Kung University, Tainan, Taiwan; 13 Department of Nursing, Chung Hwa University of Medical Technology, Tainan, Taiwan; 14 National Institute of Cancer Research, National Health Research Institutes, Miaoli, Taiwan; 15 University Center for Bioscience and Biotechnology, National Cheng Kung University, Tainan, Taiwan; 16 Department of Chemistry, National Tsing Hua University, Hsinchu, Taiwan

**Keywords:** Cathepsin S, Interleukin-7, Oral cancer, Tumor immunity, Immune checkpoint therapy

## Abstract

**Background:**

Immunomodulatory agents benefit a small percentage of patients with oral cancer (OC), a subset of head and neck cancer. Cathepsin S (CTSS), a lysosomal protease, has been frequently associated with tumor immunity. This study aimed to investigate the mechanism by which tumor CTSS affects anti-tumor immunity through the regulation of interleukin-7 (IL-7) to overcome this obstacle.

**Methods:**

OC patients’ samples were used to disclose the correlation among CTSS and CD8^+^ T cell infiltration levels. The cytokine array was used to investigate the effect of CTSS on the secretion of cytokine/chemokines. We utilized various cell biology experiments to investigate the molecular mechanism of CTSS that mediates IL-7 secretion in OC cell lines, including fluorescence resonance energy transfer, immunogold-labeled transmission electron microscopy, IL-7-enzyme-linked immunosorbent assay, immunofluorescence staining, and pull-down assay. Two syngeneic OC mice models were utilized to investigate the anti-cancer effects and the tumor immunity modulation effects of RJW-58, a CTSS activity inhibitor, and the combination with the anti-PD-1 antibody.

**Results:**

CTSS expression was inversely correlated with CD8^+^ T-cell infiltration in clinical samples. In vivo and in vitro studies using a mouse OC tumor model showed that CTSS-knockdown inhibited tumor growth and enhanced CD8^+^ T cell proliferation. These results were counteracted by co-treatment with anti-CD8 or anti-IL-7 antibodies. CTSS inhibition also remodeled the memory CD8^+^ T cell subsets within tumor tissues in vivo. Mechanistically, CTSS inhibited IL-7 secretion by disrupting its intracellular transport route. This was achieved by recognizing the intracellular domain of the IL-7 receptor (IL-7R), which bound IL-7 in granular vesicles. RJW-58 enhanced IL-7 secretion and exerted an anti-tumor effect. RJW-58 enhanced the therapeutic effect of the anti-PD-1 antibody in syngeneic mouse models.

**Conclusion:**

The findings indicate that CTSS negatively regulates IL-7 secretion by interacting with IL-7R. The CTSS-targeting strategy has the potential to reinvigorate IL-7-directed anti-tumor T cell immunity and enhance the therapeutic effect of the anti-PD-1 antibody.

**Graphical Abstract:**

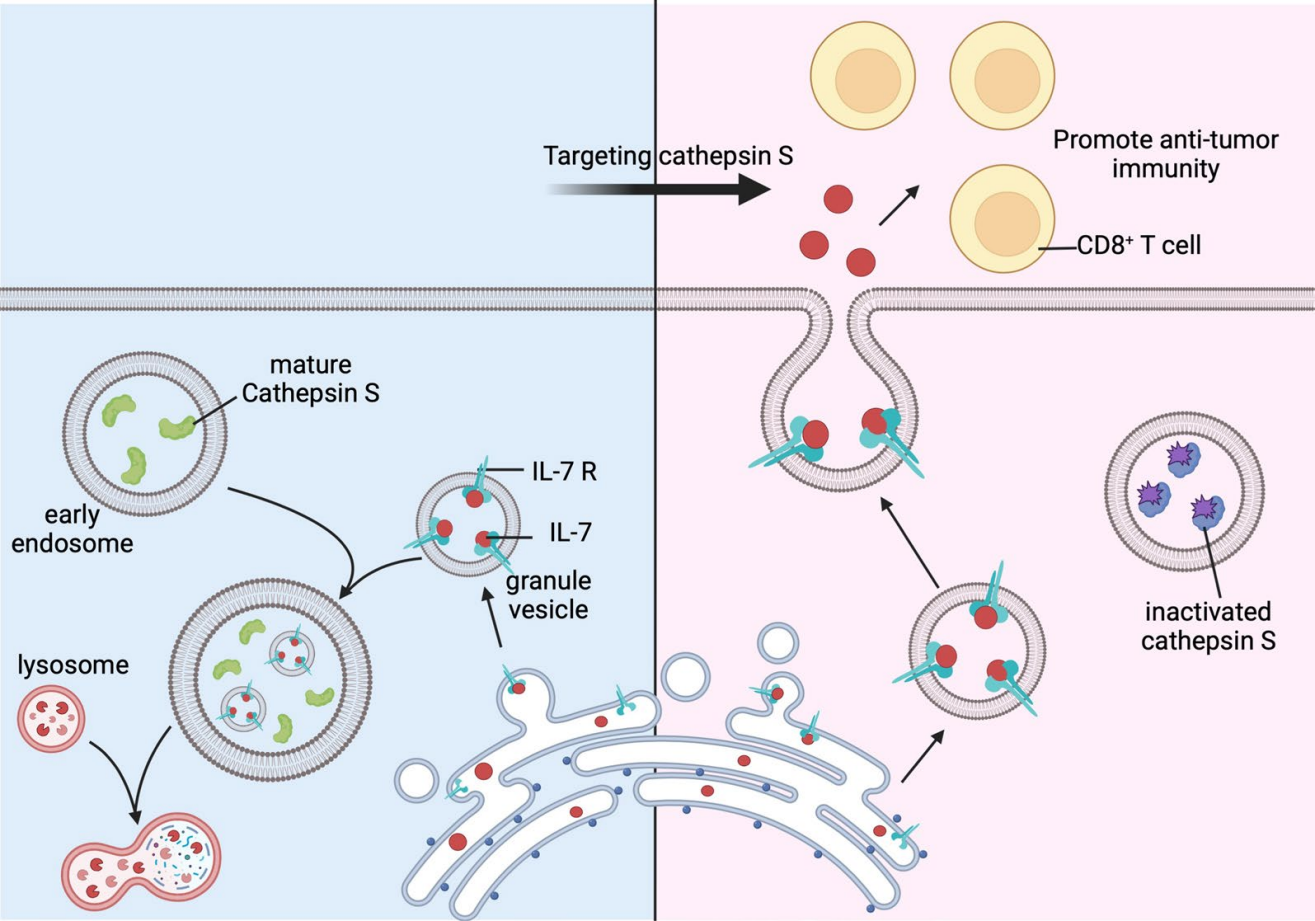

**Supplementary Information:**

The online version contains supplementary material available at 10.1186/s12929-025-01154-6.

## Background

Oral cancer (OC) is the most common subtype of head and neck cancer (HNC), and ranks as the sixth leading cause of cancer-related deaths globally [1]. Although OC has a weaker association with human papillomavirus, the tumor profiles of OC are mostly indistinguishable from those of other HNCs because of common risk factors. Squamous cell carcinoma is the most common form of this OC, which can be effectively treated with surgical resection in the early stage. In advanced stages, treatment requires a more intensive approach involving radiotherapy and chemotherapy. Despite these treatments, the general outcomes remain disappointing, with a 5-year overall survival rate of approximately 50% [2]. Recently, immune checkpoint therapies (ICTs) have provided novel therapeutic options for HNC. However, ICTs benefit only a relatively small subset of patients with OC and achieve a modest response rate (10%–20%). Multiple factors, including PD-L1 expression, tumor mutation burden, T cell-inflamed phenotypes, and T cell infiltration, have been reported to affect anti-tumor immunity in HNC [3]. Therefore, identifying potential targets that enhance the antitumor effect of ICTs is critical.

Cytokines have been proposed as potential mediators influencing the quality of lymphocytes [4]. Interleukin-7 (IL-7) plays an essential role in maintaining T cell homeostasis and functions at every stage of T cell development [5, 6]. IL-7 binds to the IL-7 receptor (IL-7R) to promote lymphocyte survival and proliferation [7]. The administration of the recombinant IL-7 protein reduced tumor burden with extensive lymphocytic infiltration of the tumor and enhance survival through the CXCR3 signaling pathway in lung cancer-bearing mice [8]. Furthermore, a phase I clinical study showed that IL-7 increased the number of peripheral CD8^+^ T cells in advanced cancer [9]. IL-7 is known to promote B-cell survival [10], enhance natural killer cell (NK cell) precursor, and increase the cytotoxic activity of NK cells [11, 12]. However, a phase I clinical trial revealed that the recombinant IL-7 increased T cells without affecting B cells and NK cells [13]. These findings indicate that IL-7 is a promising candidate for increasing the efficacy of ICTs [14]. A recent study showed that a bispecific anti-PD-1/IL-7 antibody could overcome ICTs unresponsiveness in tumors [15]. However, cytokines are often short-lived and notorious for inducing adverse events. Therefore, exploring alternative treatment approaches for ICTs-unresponsive tumors is crucial.

Cathepsin S (CTSS), a lysosomal cysteine protease, plays an important role in intracellular protein degradation. It shares common characteristics with other family members, stemming from its canonical function as an endopeptidase [16]. CTSS overexpression has been reported in various cancers, including HNC, breast cancer, and lung cancer [16, 17]. CTSS plays a crucial role in tumor progression, metastasis, and angiogenesis. Thus, it can serve as a potential therapeutic target [18, 19]. Additionally, CTSS regulates immunity by promoting major histocompatibility complex (MHC) class II to activate CD4^+^ regulatory T cells (Treg) and inhibiting MHC class I to suppress CD8^+^ T cell activity [20]. CTSS plays a pivotal role in mediating inflammatory responses by inducing the release of multiple proinflammatory cytokines [21].

Multiple CTSS-targeting agents have been developed. However, they commonly face problems with cross-interactions with other cathepsins, resulting in undesirable side effects [22, 23]. We developed a highly specific CTSS inhibitor, RJW-58, to overcome these obstacles [24, 25]. RJW-58 exhibited an anti-tumor activity and altered the expression of multiple cytokines in the serum of C57BL/6JNarl mice, with IL-7 being a significant factor [26]. This study aimed to investigate the mechanism by which CTSS mediates IL-7 secretion and its impact on anti-tumor immunity and to evaluate the potential of RJW-58 to support the anti-PD-1 antibody (αPD-1).

## Materials and methods

### Culture of human and mouse OC cell lines

Human OC cell lines, OEC-M1, SAS, and TW2.6, were used in this study [27, 28]. Normal human oral keratinocyte cell, DOK cells, was used in this study [29]. OEC-M1 cells were cultured in RPMI medium (*cat#*22400–89; ThermoFisher Scientific, Waltham, MA, USA) while SAS and TW2.6 cells were cultured in DMEM/F12 medium (*cat#*11320–033; ThermoFisher Scientific). DOK cells were cultured in DMEM (*cat#*11995–040; ThermoFisher Scientific) supplemented with 2 mM Glutamine and 5 μg/mL Hydrocortisone [30]. NHRI-HN1 was a self-developed mouse OC cell line originating from a 4-ntitroquinoline 1-oxide (4-NQO)-induced tumor that grew in the oral cavity of C57BL/6 mice, and a rapidly growing subclone was selected for use in the present study [31]. Cells were cultured in DMEM medium. MOC-1 was also a C57BL/6-originated OC cell line and was cultured in the DMEM/F12 medium. All mediums were supplemented with fetal bovine serum (*cat#*SH30396.03; Cytica, Marlborough, MA, USA) and penicillin/streptomycin (*cat#*15140–122; ThermoFisher Scientific). All cells were incubated at 37 °C in an incubator containing 5% CO_2_ in the air. All cell lines were tested negative for mycoplasma contamination.

### Tumor samples

HNC tissue array (Product Number: HN1310) was obtained from TissueArray.Com LLC (Rockville, MD, USA) (Table S1). Archival tissue of OC was obtained along with the tumor staging information from the tissue bank of Chi-Mei Medical Center (Liouying, Tainan, Taiwan) (N = 20) and National Cheng Kung University Hospital (N = 50; all were used for CTSS or CD8 staining, while 25 of them were used for IL-7R staining) by the regulation of the Independent Review Board (Table S2). Tissue blocks were obtained from OC patients who were diagnosed between January 2002 and December 2010, and had undergone curative surgery at National Cheng Kung University Hospital. No distant metastases were identified at any of the initial diagnoses. The last follow-up date was in 2014. Studies of human samples were approved by the Independent Review Board of National Health Research Institutes (EC1100212), National Cheng Kung University Hospital (B-ER-110–230), Chi Mei Medical Center (11201-L03).

### Immunohistochemistry staining (IHC)

Sliced formalin-fixed, paraffin-embedded tissue on the glass slide was deparaffinized and dehydrated through incubation in xylene and gradient ethanol (100% to 70%). They were incubated in hydrogen peroxide to block endogenous peroxidase, followed by antigen retrieval through autoclave within citrate buffer. The tissue was then stained with the corresponding primary antibodies including anti-CTSS antibody (*cat#*sc-271619; Santa Cruz, Dallas, Texas, USA), anti-IL-7 antibody (*cat#*sc-365306; Santa Cruz), anti-human CD8 antibody (*cat#*372902; BioLegend, San Diego, CA, USA), and anti-mouse CD8 antibody (*cat#*14–0808-80; ThermoFisher Scientific), and the secondary antibody (*cat#*K5007; Dako; Santa Clara, CA, USA). The immunoreactivity was detected and revealed by application of EnVision™ + kit (*cat#*4065; Agilent; Santa Clara, CA, USA). Hematoxylin (*cat#*ab220365; Abcam, Cambridge, UK) was used for counterstaining of the nucleus. To quantify, the expression of CTSS and IL-7 were calculated by using H-score method [H-score = Σ*Pi* (*i* + 1). *Pi* represents the percentage of tumor cells stained at respective intensities (0%–100%), and *i* represents for tumor-staining intensity (0–3 +)] [32]. The infiltration of CD8^+^ T cells were scored based on the density [1 = no or sporadic (0–10 CD8^+^ T cells), 2 = moderate (11–30 CD8^+^ T cells), 3 = abundant (31–50 CD8^+^ T cells), 4 = highly abundant infiltration (> 50 CD8^+^ T cells)] [33]. A total CD8 score ranging from 3 to 12 was calculated upon the sum of the single CD8 expression level of every subsite.

### Cytokine array analysis

Serum was collected from the mice, which were implanted with NHRI-HN1 cells carrying either sh*Ctss* or shControl, after tumor inoculation for 18 days. Cytokine array analysis was performed following the protocol of the MILLIPLEX Mouse High Sensitivity T cell Magnetic Bead Panel (cat#MHSTCMMAG-70 K; Millipore, Burlington, MA, USA). The cytokine levels were measured by the Luminex^®^ 200^™^ with the xPONENT^®^ software.

### RNA-based gene modulation

siRNA including transfecting control siRNA-A (a non-targeting control siRNA) (*cat#*sc-37007; Santa Cruz), human CTSS siRNA (h) (*cat#*sc-29940; Santa Cruz), mouse CTSS siRNA (m) (*cat#*sc-29941; Santa Cruz), human CTSB siRNA (h) (*cat#*sc-29238; Santa Cruz), human CTSL siRNA (h) (*cat#*sc-29938; Santa Cruz), human IL-7 receptor siRNA (h) (*cat#*sc-35664; Santa Cruz), human LAMP-1 siRNA (h) (*cat#*sc-29389; Santa Cruz), human IL-2Rγ siRNA (h) (*cat#*sc-35653; Santa Cruz), siGENOME Non-targeting siRNA pool#1 (*cat#*D-001206–13-05; Dharmacon, Lafayette, CO, USA), or siGENOME Human IL7R (3575) siRNA–SMARTpool (*cat#*M-007996–01-0005; Dharmacon) was used to knockdown interested genes in OC cell lines. After cell adhesion, Lipofectamine™ RNAiMAX (*cat#*56532; ThermoFisher Scientific) and siRNA were separately diluted in the OPTI-MEM I medium. They were then mixed at 1:1 ratio and incubated for 20 min at room temperature. After the preparation process, the transfection mixture was loaded onto cells that were incubated in the antibiotic-free culture medium for the indicated time.

### Isolation and stimulation of mouse CD8^+^ T cells

The spleen of C57BL/6 mice was taken and minced in PBS. After incubating with RBC lysis kit, splenocytes were collected through passing the strainer. The CD8a^+^ T cell isolation kit (*Cat#*130–104-075; Miltenyi Biotec, Bergish, Gladbach, Germany) was applied following the Manufacturer’s protocol for isolation of CD8^+^ T cells. Isolated T cells were then cultured in the serum and antibiotic-containing RPMI medium. Subsequently, isolated CD8^+^ T cells were stimulated with the Dynabeads Mouse T-Activator CD3/CD28 (cat#11452D; ThermoFisher Scientific) and 30U/mL recombinant interleukin-2 (IL-2) (cat#212–12; ThermoFisher Scientific).

### Water-soluble tetrazolium (WST) cell proliferation assay

Dynabeads Mouse T-Activator CD3/CD28 and IL-2-stimulated mouse CD8^+^ T cells were treated with the conditioned medium (CM), which was collected from the Ctss-knockdown NHRI-HN1 or MOC-1 cells for 48 h. After treatment, CD8^+^ T cells were then incubated with WST, Sodium 4-(2-(4-iodophenyl)-3-(4-nitrophenyl)tetrazol-2-ium-5-yl]benzene-1,3-disulfonate (*cat#*MK400; Takara, Kyoto, Japan) for 30 min, and the cell proliferation was analyzed by measuring the absorbance of the solution at 440 nm wavelength by the Sunrise™ absorbance microplate reader.

### Carboxyfluorescein succinimidyl ester (CFSE) cell proliferation assay

Isolated CD8^+^ T cells were stained with the cellTraceTM CFSE cell proliferation Kit (*cat#*C34554; ThermoFisher Scientific) at 37℃ for 30 min, followed by activation with the Dynabeads Mouse T-Activator CD3/CD28 and 30U/mL recombinant IL-2. Stimulated CD8^+^ T cells were treated with NHRI-HN1 or MOC-1 CM for 48 h. After treatment, the CFSE intensity was analyzed by flow cytometry (Attune Nxt, ThermoFisher Scientific).

### RNA extraction and quantitative real-time PCR (qPCR).

Total RNA was extracted from culture cells by using TRIzol^TM^ Reagent (*cat#*15596018; ThermoFisher Scientific), and was then synthesized to cDNA by using the Maxima First Strand cDNA synthesis Kit (*cat#*K1642; ThermoFisher Scientific). Equal amount of the cDNA product was then loaded with primers and the SYBR^TM^ Green Master Mix (*cat#*4385612; ThermoFisher Scientific) for qPCR amplification by using the ABI 7000 Sequence Detection System. The target fragment was amplified using specific primers listed in the following: *human CTSS* forward:3’-CTCTTGGTGTGCTCCTCTG-5’; *human CTSS* reverse:3’-CGTCGTACTGCTTCTTCATTC-5’; *human GAPDH* forward:3’-TGCACCACCAACTGCTTAGC-5’; *human GAPDH* reverse:3’-GGCATGGACTGTGGTCATGAG-5’. The expression fold change of target genes was read during the process and quantified using comparative threshold cycle 2^−ΔΔCt^ method (ΔCt = Ct_Target_ _gene_-Ct_*GAPDH*_, ΔΔCt = ΔCt_Treatment_-ΔCt_Control_).

### Enzyme-linked immunosorbent assay (ELISA) for detection of IL-7

The human IL-7 ELISA Kit (*cat #*EHIL7; ThermoFisher Scientific) was applied to detect and quantify the secreted IL-7 by OC cells. Briefly, cells were seeded onto each well of 96-well plates with the number of 2 × 10^4^ cells/well for 16 h before transient transfection, sucrose, Bafilomycin A1 (BafA1), curcumin, Z-Phe-Tyr-CHO, E-64, or RJW-58 treatment for another 72 h. After procedures, the cultured medium was transferred to a 96-well microplate with the indicated concentration of anti-IL-7 antibody added into each well. Later, the microplate was incubated with 3, 3’, 5, 5’-tetramethylbenzidine for 30 min at room temperature, followed by measuring the absorbance of the solution at 450 nm wavelength by TECAN reader to quantify the IL-7 amount. For detect the serum IL-7 level, the mouse IL-7 ELISA Kit (*cat #*EMIL7; ThermoFisher Scientific) was applied to detect and quantify the IL-7 in mice serum. Briefly, serum was transferred to a 96-well microplate with the indicated concentration of anti-IL-7 antibody added into each well. Later, the microplate was incubated with 3, 3’, 5, 5’-tetramethylbenzidine for 30 min at room temperature, followed by measuring the absorbance of the solution at 450 nm wavelength by TECAN reader to quantify the IL-7 amount.

### Transfection of DNA plasmids

pCMV6-AC-CTSS (*cat#*sc319097; Origene, Rockville, MD, USA), pCMV6-AC (*cat#*ps100020; Origene), mouse CTSS shRNA plasmid (m) (*cat#s*c-29941-SH; Santa Cruz), and control shRNA plasmid-A (a control plasmid DNA that expresses non-targeting shRNA) (*cat#*sc-108060; Santa Cruz) were purchased from commercial companies. Mouse CTSS shRNA plasmid (m) and control shRNA plasmid-A was used for the stable CTSS-knockdown NHRI-HN1 selection. pCDNA3.1 + C-eGFP-IL-7, pmRFP-C3-V-SNARE, pCDNA3.1 myc-IL-7R, pCDNA3.1 myc-IL-7R 1–414, pCDNA3.1 myc-IL-7R 1–364, pCDNA3.1 myc-IL-7R 1–314, pCDNA3.1 myc-IL-7R 1–264, pCDNA3.1 myc-IL-7R 427AA, pCDNA3.1 myc-IL-7R 433AA, pCDNA3.1 myc-IL-7R 445AA, pCDNA3.1 myc-IL-7R 448AA, pCMV6-AC-mGFP-CTSS, pCMV6-AC-mRFP-IL-7R, and pCMV6-AC-mRFP-IL-7R 1–264 were synthesized by the Protech Technology Enterprise Company, Taipei, Taiwan. All plasmid DNA were purified by using the QIAGEN plasmid Midi Kit (*cat#12145*; QIAGEN, Hilden, Germany). Then, Lipofectamine^™^ LTX reagent with PLUS^™^ reagent (*cat#*15338,100; ThermoFisher Scientific) was used to transfect DNA plasmids into OEC-M1, SAS, TW2.6, and NHRI-HN1 cells. After cell adhesion, Lipofectamine^™^ LTX was diluted in the OPTI-MEM I medium. Purified plasmid DNA were diluted in the OPTI-MEM I medium to be mixed with Lipofectamine PLUS™ reagent. The plasmid-DNA-PLUS mixture was then mixed with the Lipofectamine LTX™ reagent in a ration of 1:1 ratio for 20-min incubation at room temperature. The final product was then loaded onto the cells under antibiotic-free culture medium and incubated for indicated durations.

### Time-lapse immunofluorescent microscopy

OEC-M1 cells were first co-transfected with IL-7-GFP/V-SNARE-RFP-expressed plasmid or IL-7-GFP/IL-7R-mRFP-expressed plasmid then transfected with scramble siRNA and *CTSS* siRNA for another 48 h. After transfection, OEC-M1 cells were imaged every 30 s for 30 min with the live cell fluorescent inverted microscope with LAS X software (cat#DMi8; Leica, Wetzlar, Germany).

### Immunofluorescent confocal microscopy

OEC-M1 cells were seeded onto glass coverslips for 16 h. For fixation, they were incubated with 4% paraformaldehyde at room temperature for 15 min. After washing with PBS, cells were permeabilized with PBST (PBS containing 1% Triton X-100) at room temperature for 30 min. Subsequently, permeabilized cells were incubated with blocking solution (5% BSA (cat#A9647; Sigma Aldrich)) at room temperature for 1 h, then with primary antibodies (anti-CTSS antibody (*cat#*sc-271619; Santa Cruz), anti-Rab5 antibody (*cat#*GTX636971; GeneTex, Irvine, CA, USA), anti-IL-7 receptor antibody (*cat#*GTX54311; GeneTex), anti-LAMP1 antibody (*cat#*GTX33293; GeneTex), anti-GOLGA5 antibody (*cat#*GTX104255; GeneTex), anti-E-cadherin antibody (*cat#*629692; GeneTex), anti-Calnexin antibody (*cat#*629976; GeneTex), and anti-myc-tag antibody (*cat#*2276; Cell signaling, Danvers, MA, USA)) at 4℃ overnight. Later, after PBST washing, cells were incubated with secondary antibodies at room temperature for 1 h. They were then incubated with immunotag™ VTI1B (V-SNARE) antibody-Alexa fluor 647 (*cat#*ITA5488; G-Biosciences; Louis, MO, USA), Goat anti-Rabbit IgG (H + L)-Alexa Fluor 594 (*cat#*A11012, ThermoFisher Scientific), Goat anti-Mouse IgG (H + L)-Alexa Fluor 488 (*cat#*A11001, ThermoFisher Scientific), Goat anti-Mouse IgG (H + L)-Alexa Fluor 546 (*cat#*A11030, ThermoFisher Scientific), and Chicken anti-Mouse IgG (H + L)-Alexa Fluor 594 (*cat#*A21201, ThermoFisher Scientific) at room temperature for 1 h before mounted with glycerol-gelatin. Nuclei were counter-stained with ProLong Gold antifade reagent with DAPI (*cat#*36935; ThermoFisher Scientific). The images were taken by Fluoview FV300 scanning confocal microscope (Olympus, Hachioji, Tokyo, Japan).

### Cytoplasmic protein extraction

OEC-M1 cells were seeded onto 10 cm dish and incubated overnight. Cytoplasmic proteins were extracted with the Nuclear complex Co-IP Kit (cat#54001; Active Motif, Rixensart, Belgian). Briefly, cells were washed with ice-cold PBS and was scrapped from dishes. After centrifugation, cell pellet was incubated the Hypotonic Buffer 15 min, then detergent was added. After centrifugation, the supernatant (cytoplasmic fraction) was collected and applied to the immunoprecipitation assay.

### The immunoprecipitation assay (IP)

Cells were seeded onto 10 cm dishes at the number of 1 × 10^6^ and incubated overnight. Then cells were transfected with scramble siRNA, *CTSS* siRNA, pCDNA3.1 myc-IL-7R, pCDNA3.1 myc-IL-7R 1–364, pCDNA3.1 myc-IL-7R 1–314, or pCDNA3.1 myc-IL-7R 1–264 for 48 h. After treatment, cells were harvested in lysis buffer containing protease inhibitor (*cat#*04693132001; Roche, Basel, Switzerland), 1 mM Na_3_VO_4_, 0.5% NP-40 (*cat#*492016-500ML; Sigma Aldrich), 10% glycerol and then fragmented by sonication on ice. Cell lysate was collected after centrifugation. 250 μg lysate was incubated with 0.5 μg mouse IgG isotype control antibody (*cat#*GTX35009; GenTex), anti-CTSS antibody (*cat#*sc-271619; Santa Cruz), anti-IL-7 antibody (*cat#*sc-365306; Santa Cruz), or anti-myc tag antibody (*cat#*sc-47694; Santa Cruz). Subsequently, lysate and antibodies mixture were incubated with pre-cleaned protein A Sepharose bead at 4 ℃ overnight. Unbound proteins and antibodies were removed after centrifugation. Then beads were washed with wash buffer (0.5% NP-40 and 0.1% Triton X-100 in PBS) three times. Proteins were eluted by incubating beads at 95℃ for 10 min.

### Western blot analysis

Cells were lysed with the RIPA buffer (*cat#*20–188; Millipore) containing protease inhibitor (*cat#*04693132001; Roche, Basel, Switzerland). Insoluble protein and membrane protein were discarded by centrifugation. Sodium dodecyl sulfate–polyacrylamide gel electrophoresis was used to separate cell lysates by the molecular weight of proteins. Subsequently, proteins were transferred onto polyvinylidene difluoride membranes (*cat#*IPVH85R; Millipore). Then membranes were blocked with 5% nonfat milk and incubated with primary antibodies (anti-CTSS antibody (*cat#*sc-271619; Santa Cruz), anti-IL-7 antibody (*cat#*sc-365306; Santa Cruz), anti-IL-7R antibody (*cat#*GTX54311; GeneTex), anti-myc-tag antibody (*cat#*2276; Cell signaling), anti-LAMP1 antibody (*cat#*GTX33293; GeneTex), anti-CTSB antibody (cat#A19005; ABcloncal, Woburn, MA, USA), anti-CTSL antibody (cat#A23375; ABcloncal), anti-GAPDH antibody (*cat#*sc-32233; Santa Cruz), and anti-β-actin antibody (*cat#*MAB1501; Millipore)). After primary antibodies incubation, membranes were incubated with secondary antibodies (anti-Mouse IgG antibody (*cat#*AP124P; Sigma) and anti-Rabbit IgG antibody (*cat#*AP132P; Sigma)) at room temperature for 1 h. A chemiluminescence substrate (*cat#*WBLUR0500; Millipore) was used to elicit signals for the detection of the intensity. The chemiluminescence was captured with the photographic film (cat# C3F427700, FujiFilm, Tokyo, Japan).

### Transmission electron microscopy (TEM) of treated cells for immunogold labelling

The study applies the previous method as follow [34]. The cells were collected and fixed in a fixative containing 4% paraformaldehyde, 0.2% glutaraldehyde, and 3 mM CaCl_2_ in 0.1 M cacodylate buffer for 1 h at 4 °C. After washing with 0.1 M cacodylate buffer containing 3 mM CaCl_2_, cells were post-fixed for 1 h at 4 °C in 0.1 M cacodylate buffer containing 1% osmium tetroxide and 1.5% potassium ferricyanide. Samples were washed with distilled water, followed by gradual dehydration in a graded ethanol series of 70%, 90%, 95% (15 min per stage), and then 100% (three times and 30 min each time). The dehydrated samples were then infiltrated in stages with spur resin–ethanol solutions containing 50%, 75%, and 100% resin (1 h per stage). The infiltrated samples were left in 100% spurr resin overnight. Next, samples were embedded in the models with fresh resin and polymerized at 70 °C for 24 h. The embedded cells were cut using an ultramicrotome (Ultracut S, Leica Reichart) into 70 nm ultrathin sections using a diamond knife and harvested on nickel grids (*cat#* FF100-Ni; Electron Microscopy Science). The thin sections were first treated with 10% H_2_O_2_ in buffer for 10 min. The rinsed thin sections were then floating on the blocking solution (5% FBS) for 1 h and then incubated with primary antibodies (anti-IL-7 antibody (*cat#*sc-365306; Santa Cruz) and anti-IL-7R antibody (*cat#* GTX54311; GeneTex)) for 2 h at 37 °C. The thin sections were washed with PBST and incubated in 1% BSA for 10 min. After then, the thin sections were incubated with secondary antibody, including Goat anti-mouse IgG (*cat#*115–405-166; Jackson ImmunoResearch) and Goat anti-rabbit IgG (*cat#*ab270555; Abcam) coupled to 40 nm and 10 nm gold particles, respectively. The thin sections were washed with PBST and incubated with 4% paraformaldehyde for 10 min. The thin sections were post-stained with uranyl acetate and lead citrate, and then examined by JEOL-1400 electron microscope (JEOL, Tokyo, Japan) at 120 keV equipped with CCD Camera System (Ultrascan, Gatan, Pleasanton, CA, USA).

### CTSS hydrolysis assay

Recombinant IL-7R (7200 ng) (*cat#*TP309687; Origene) was incubated with 60 mg CTSS (*cat#*1183-CY-010; Bio-Techne, Minneapolis, MN, USA) in a reaction buffer (100 mM MES (pH 6.0), 16 mM dithiothreitol, and 1.6 mM ethylenediaminetetraacetic acid (EDTA)) at 37 °C for 0–30 min. The reacted samples were separated by the SDS-PAGE. After the gel electrophoresis, the SDS-PAGE was incubated in the 0.1% Coomassie blue staining solution in room temperature for 30 min. Subsequently, the SDS-PAGE was incubated with the destaining solution (40% methanol and 10% glacial acetic acid) until the background was destained. In addition, the western blot analysis was applied to analysed the reacted samples with the anti-IL-7R antibodies.

### Fluorescence resonance energy transfer assay (FRET) for IL-7R and IL-7 protein–protein interaction

Cells were seeded onto the 3.5 cm dishes with cover slides at the number of 10 × 10^4^ and incubated overnight. Then cells were co-transfected with pCMV6-AC-mGFP-CTSS (as a donor, excitation/emission: 483 and 506 nm) and pCMV6-AC-mRFP-IL-7R or pCMV6-AC-mRFP-IL-7R 1–264 (as an acceptor, excitation/emission: 555 and 584 nm) by Lipofectamine™ LTX reagent with PLUS™ reagent and treated for another 48 h. Cells were fixed and nuclei were counter-stained with ProLong Gold antifade reagent with DAPI (*cat#*36935; ThermoFisher Scientific). The images were taken by the FV1000 scanning confocal microscope (Olympus).

### CTSS activity assay

Cells were seeded onto 6 cm dishes at the number of 3 × 10^5^ and incubated overnight. Then cells were transfected with CTSS siRNA or treated with RJW-58 for 48 h. The CTSS activity was analyzed with the CTSS activity assay kit (cat#ab65307; Abcam). Cells were lysed in the CTSS lysis buffer. Then supernatant was incubated with reaction buffer and CTSS substrate. The CTSS activity was analyzed by measuring the absorbance at 500 nm by the CLARIOstar (BMG LABTECH, Offenburg, Germany).

### Construction of syngeneic mouse model and the animal experiments

Animal studies were approved by the Institutional Animal Care and Use Committee of National Health Research Institutes (110017 and 113052). Considering the sexual disparity with a tendency of more activated CD8^+^ T-cells against tumor than male mice, we used only female mice to create a more immune-relevant microenvironment [35]. NHRI-HN1, scramble NHRI-HN1, and shCtss-NHRI-HN1 cells were injected subcutaneously into the flank area at 1 × 10^6^ in 8–9-week-old C57BL/6 mice. MOC1 cells were injected subcutaneously into the flank area at 3 × 10^6^ in 8–9-week-old C57BL/6 mice. Mice were randomly assigned for treatment after tumorinoculation. For antibodies applied in this study were given by intraperitoneal injection twice a week. Antibodies were listed in the following: Polyclonal Armenian rat IgG2 isotype control (10 μg/mice) (*cat#*BE0086; BioXcell, Lebanon, NH, USA), Anti-mouse/human IL-7 antibody (αIL-7) (10 μg/mice) (*cat#*BE0048; BioXcell), Polyclonal Armenian hamster IgG (100 μg/mice) (*cat#*BE0091; BioXcell), αPD-1 (100 μg/mice) (*cat#*BE0033-2; BioXcell), Rat IgG1 isotype control, anti-horseradish peroxidase (200 μg/mice) (*cat#*BP0088; BioXcell), Anti-mouse CD8b antibody (Lyt3.2) (αCD8) (200 μg/mice) (*cat#*BE0223; BioXcell). For RJW-58, the compound was given by intraperitoneal injection 5 days a week. Tumor size and body weight were measured every two days. The tumor size was calculated as the following formula: tumor size = (Length × Width^2^) × 0.5. At the end of the study or once the animal reached the humane endpoint, mice were euthanized. Tumor, spleen, and blood were immediately collected for further analysis. All data were collected except mice died before the end of the experiments. The attrition rate in our study was less than 1%.

### Tumor dissociation assay

Infiltrated immune cells were isolated from the tumor with the Tumor Dissociation kit (cat#130–096-730; Miltenyi Biotec, Bergisch Gladbach, Germanny). Harvested mice tumors were fragmented and incubated in an RPMI-based enzyme master mixture. The tumor tissue-enzyme master mixtures were loaded into the gentleMACS C Tube and applied to the gentleMACS^TM^ Octo Dissociator in the 37C_m_TDK_1 program. After the program, samples were resuspended in the RPMI medium containing 5% FBS, MEM Non-essential amino acids (*cat#*11140–050; ThermoFisher Scientific), sodium pyruvate (*cat#*11360–070; ThermoFisher Scientific), and β-ME and transferred to the Pre-separation Filter with the 70 μM pore size (cat#130–095-823; Miltenyi Biotec). The flow through was collected with tubes. After centrifugation, cell pellet was resuspended and incubated in ACK lysis buffer (*cat#*BP10-548E; Lonza, Basel, Switzerland) at room temperature for 5 min. Subsequently, RPMI medium was added and applied to centrifugation. Then cell pellets were resuspended in the 80% percoll^TM^ solution (*cat#*17089101; Cytiva, Washington, D.C., USA), gently added the 40% percoll^TM^ solution into tubes, and centrifuged at room temperature for 20 min without braker. The leukocytes layer was collected to new centrifuge tube, mixed with RPMI medium, and centrifuged. After centrifugation, cell pellets were resuspended in RPMI medium and were stimulated with the eBioscience^TM^ Cell Stimulation Cocktail (*cat#*00–4975-93; ThermoFisher Scientific) at 37℃ for 5 h.

### Lysosome isolation assay

The Lysosome isolation Kit (*cat#*LYSIS01; Sigma Aldrich) was applied to detect the protein expression patent in the OC cells. Briefly, 3 × 10^8^ cells were collected by trypsinization. Cells were incubated with the Extraction buffer and were broken with homogenizer. After centrifugation, the supernatant was collected as the cytoplasmic fraction. The pellet was resuspended in the Extraction buffer as a Crude lysosome fraction. The crude lysosome fraction was added to the 19% OptiPrep^TM^ Density Gradient Medium Solution. Then, 8 mM CaCl_2_ was added and centrifuged at 5000 g for 15 min. The supernatant was collected as the lysosome fraction. All fractions were applied to the Western blotting analysis.

### Fluorescence activated cell sorting assay (FACS)

The FACS assay was used to analyze the changes of lymphocytes, including CD8^+^ T cells, activated CD8^+^ T cells, exhausted CD8^+^ T cells, regulatory CD8^+^ T cells (CD8 Treg), Naïve CD8^+^ T cells, central memory CD8^+^ T cells (CD8 Tcm), effector memory CD8^+^ T cells (CD8 Tem), tissue-resident memory CD8^+^ T cells (CD8 Trm), CD4^+^ T cells, regulatory CD4^+^ T cells (CD4 Treg), central memory CD4^+^ T cells (CD4 Tcm), effector memory CD4^+^ T cells (CD4 Tem), and tissue-resident memory CD4^+^ T cells (CD4 Trm) in CTSS-targeted syngeneic OC mouse model. The stimulated mice immune cells were labeled with fluorescent-conjugated antibodies (anti-mouse CD45 antibody (*cat#*103138; BioLegend, San Diego, CA, USA), anti-mouse CD3 antibody (*cat#*100215; BioLegend), anti-mouse CD4 antibody (*cat#*11–0042-82; ThermoFisher Scientific), anti-mouse CD8 antibody (*cat#*100734; BioLegend), anti-mouse CD279(PD-1) antibody (*cat#*25–9985-82; ThermoFisher Scientific), anti-mouse CD25 antibody (*cat#562694*; BD biosciences, Franklin Lakes, NJ, USA), anti-mouse FOXP3 antibody (*cat#*17–5773-82; ThermoFisher Scientific), anti-mouse CD45RA antibody (*cat#752984*; BD biosciences), or anti-mouse CD62L antibody (*cat#*104428; BioLegend), anti-granzyme B antibody (*cat#*12–8898-82; ThermoFisher Scientific), anti-CD44 antibody (*cat#563971*; BD biosciences), anti-CCR7 antibody (*cat#*47–1971-82; ThermoFisher Scientific)), which recognized cell surface protein at room temperature for 15 min. The antibodies-labeled cells were washed and fixed/permeabilized at 4℃ for 16 h. After fixation and permeabilization, cells were washed and labeled with fluorescent-conjugated antibodies, which recognized cytoplasmic protein at room temperature for 30 min. Subsequently, cells were collected by centrifugation, resuspended in PBS and applied to the flow cytometry (Attune Nxt, ThermoFisher Scientific). The gating strategy was shown in Figure S3.

### Statistical analysis

Each experiment was repeated at least three times. Data are presented as mean ± SEM. Statistical tests were performed using GraphPad Prism10. Two-tailed Student *t*-test was used when comparing the two group. If more than two groups were compared, one-way ANOVA analysis was performed with the Tukey’s multiple comparisons test. Comparisons of tumor growth and body weight were analyzed by the two-way ANOVA analysis was performed with the Tukey’s multiple comparisons test. Because this was a pilot study, a formal power calculation was not required. The Spearman r correlation analysis was used to analyze the correlation between CTSS, CD8, and IL-7R IHC score in the head and neck cancer patients’ samples.

## Results

### Inverse correlation between CTSS and CD8^+^ T-cell infiltration in OC samples.

**Fig. 1:**
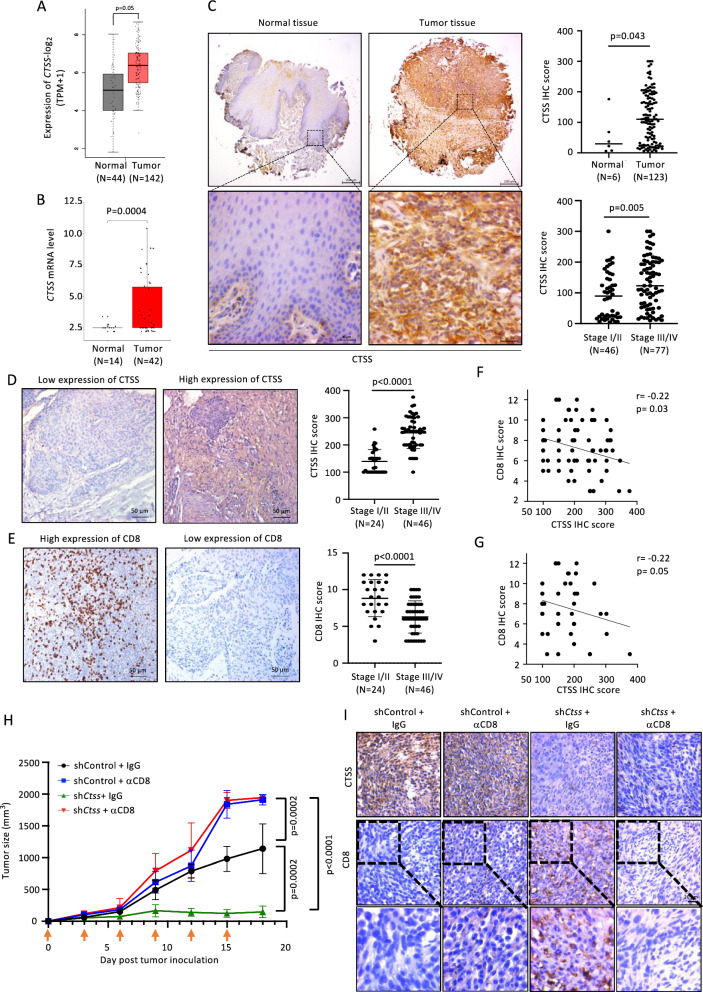
Overexpression of CTSS in HNC tissue is inversely correlated with CD8^+^ T-cell infiltration. **A** & **B** The dot graphs show the mRNA expression levels of *CTSS* in the head and neck cancer (HNC) datasets from (**A**) TCGA and (**B**) GEO (GSE6791). **C** The representative photos of the CTSS immunohistochemical (IHC) staining on the HNC tissue array. The dot graphs summarize the quantitative score of each sample by stratification. **D** & **E** The representative photos of IHC staining for (**D**) CTSS expression and (**E**) CD8^+^ T-cell infiltration of the in-house oral cancer (OC) samples were shown (N = 70). The score of each sample is plotted by different parameters on the right panels. **F** & **G** The correlation between the IHC staining score of CTSS and CD8 in the in-house OC samples was studied. The results are shown by dot graph plots for (**F**) the entire cohort (N = 70) and (**G**) samples with T1 and T2 Stage (N = 38). **H** The tumor volume of the subcutaneously-inoculated NHRI-HN1 cell is plotted. Mice were implanted with tumors carrying either sh*Ctss* or shControl, and were grouped by treatment with αCD8 or IgG antibody (N = 6 for each group). Arrowhead indicates days for antibody administration. **I** The representative photos of the IHC staining for CTSS and CD8^+^ T cells for each treatment group

The analysis of two HNC databases, The Cancer Genome Atlas Program and the Gene Expression Omnibus (GEO No. GSE6791), revealed that the mRNA level of *CTSS* in tumor tissues was higher than that in normal tissues **(**Fig. 1A–B**)**. Furthermore, the protein level of CTSS was analyzed by IHC staining of the HNC tissue array (Table S1). The CTSS level was significantly higher in tumor tissue than in normal tissue (*p* = 0.043) **(**Fig. 1C**)**. Additionally, the CTSS level was significantly higher in stage III/IV tumors than in stage I/II tumors (*p* = 0.005). In our in-house OC patient samples (*n* = 70), a higher CTSS level and a lower infiltration of CD8^+^ T cells were observed in stage III/IV tumors than in stage I/II tumors (Table S2 and Fig. 1D–E). Furthermore, the score of infiltrative CD8^+^ T cells was inversely correlated with CTSS score in the entire cohort and in the cases with early T stage (T1 and T2) (Fig. 1F and G).

A syngeneic mouse model was developed with the implantation of the 4-NQO-induced mouse OC cell line NHRI-HN1 to confirm the inhibitory role of CTSS in anti-tumor T cells [31]. The tumor growth rate of CTSS*-*knockdown NHRI-HN1 cells was significantly inhibited. However, when treated with the αCD8, the sh*Ctss* tumor growth level was unaffected and was comparable to that of the shControl plus αCD8 group **(**Fig. 1H and S1A). The IHC results showed increased CD8^+^ T-cell infiltration in Ctss*-*knockdown tumor tissues** (**F﻿i﻿g. 1I**)**. These effects were counteracted by the administration of the αCD8.

### CTSS inhibits the proliferation of CD8^+^ T cells via IL-7 in OC cells

**Fig. 2:**
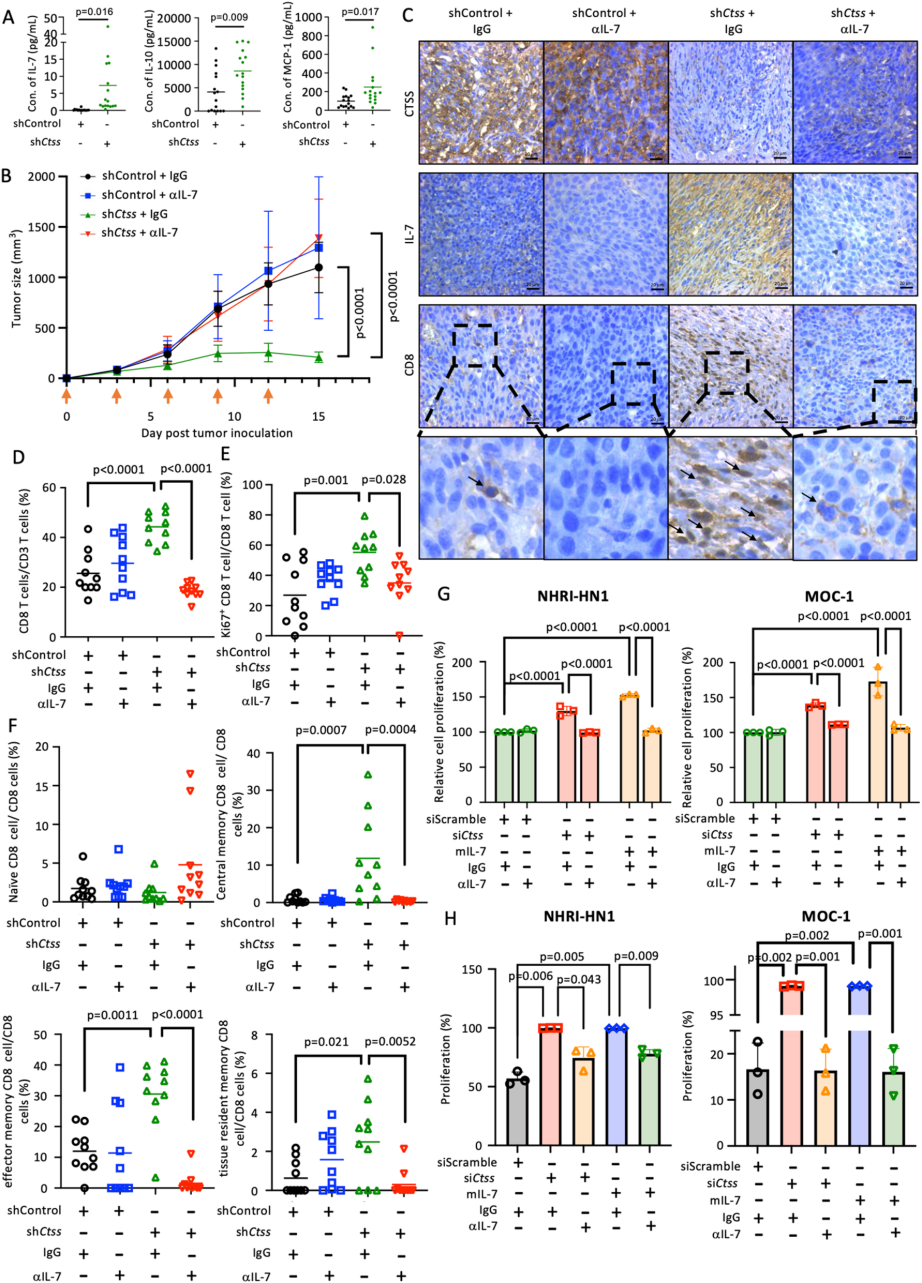
CTSS inhibits the CD8^+^ T-cells infiltration and proliferation by downregulating IL-7. **A** The expression level of IL-7, IL-10, and MCP1 is plotted in the dot graph by each sample (N = 16 in each group). **B** The tumor volume of the subcutaneously-inoculated NHRI-HN1 cell is plotted. Mice were implanted with tumors carrying either sh*Ctss* or shControl, and were grouped by treatment with αIL-7 or IgG antibody (N = 10 for each group). Arrowhead indicates days for antibody administration. **C** The representative photos of the IHC staining for CTSS, IL-7, and CD8^+^ T-cells for each treatment group. **D** Tumor infiltrative leukocytes were isolated and analyzed by FACS, the percentage of CD8^+^ cells in the target quadrant is plotted in the dot graph by each sample. **E** The percentage of Ki67^+^/CD8^+^ cells in the target quadrant is plotted in the dot graph by each sample. **F** The percentage of naïve CD8^+^ cells, central memory CD8^+^ cells, effector memory CD8^+^ cells, and tissue-resident CD8^+^ cells in the target quadrant is plotted in the dot graph by each sample. **G** & **H** Mouse CD8^+^ T-cells were treated with CM with or without the αIL-7. The CM was obtained from in vitro NHRI-HN1 or MOC-1 that were pretreated with siScramble or si*Ctss*, or that with co-incubation of the mouse IL-7 recombinant protein (mIL-7). (**G)** The result of the WST proliferation test for CD8^+^ T-cells is shown in the dot-bar graph. (**H**) The indicated scale for CFSE cell proliferation is measured and plotted in the dot-bar graph

IL-7 was the most increased cytokine, with a 10.3-fold change in the serum of the Ctss*-*knockdown NHRI-HN1 mouse model **(**Fig. 2A and S1B). Treatment with αIL-7 tended to increase tumor growth, as evidenced by the recovery of proliferation rates that had been markedly inhibited by ctss knockdown **(**Fig. 2B and S1C). Molecular studies on tumor tissues showed an increase in the local expression of IL-7 and the number of CD8^+^ T cells in the CTSS-knockdown group, which was counteracted by αIL-7 **(**Fig. 2C and S2). FACS analysis of the tumor-infiltrative leukocytes revealed that the ratio of CD3^+^/CD45^+^, CD8^+^/CD3^+^, or Ki67^+^/CD8^+^ was significantly higher in the Ctss-knockdown group than in the other groups (Fig. S3, S4, and 2D-E). Further analysis of these CD8^+^ T cells indicated enrichment of memory CD8^+^ T cells in the central memory subset (Tcm), effector memory subsets (Tem), and tissue-resident memory subsets (Trm) in Ctss-knockdown tumors (Fig. 2F) [36–38]. No statistical differences were observed in CD4^+^ T cells, CD4^+^ T cell subsets, granzyme B^+^/CD8^+^ cells, PD-1^+^/CD8^+^ cells, regulatory CD8^+^ T cells (CD8^+^ Treg), effector CD8^+^ T cells, and naïve CD8^+^ T cells (Fig. 2F and S4).

The functional relevance of CTSS and IL-7 in enhancing CD8^+^ T cell proliferation was then assessed. The CM from the si*Ctss*-transfected cells promoted the proliferation of activated mouse CD8^+^ T cells, and this effect was reversed by co-incubation with αIL-7 **(**Fig. 2G**)**. Consistently, the CFSE assay showed enhanced cell division of CD8^+^ T cells treated with CM from the si*Ctss*-transfected cells and directly stimulated with recombinant IL-7 protein. This effect was also counteracted when co-treated with the αIL-7 under both conditions **(**Fig. 2H**)**.

### CTSS negatively regulates the intracellular transport and secretion of IL-7 through IL-7R

**Fig. 3:**
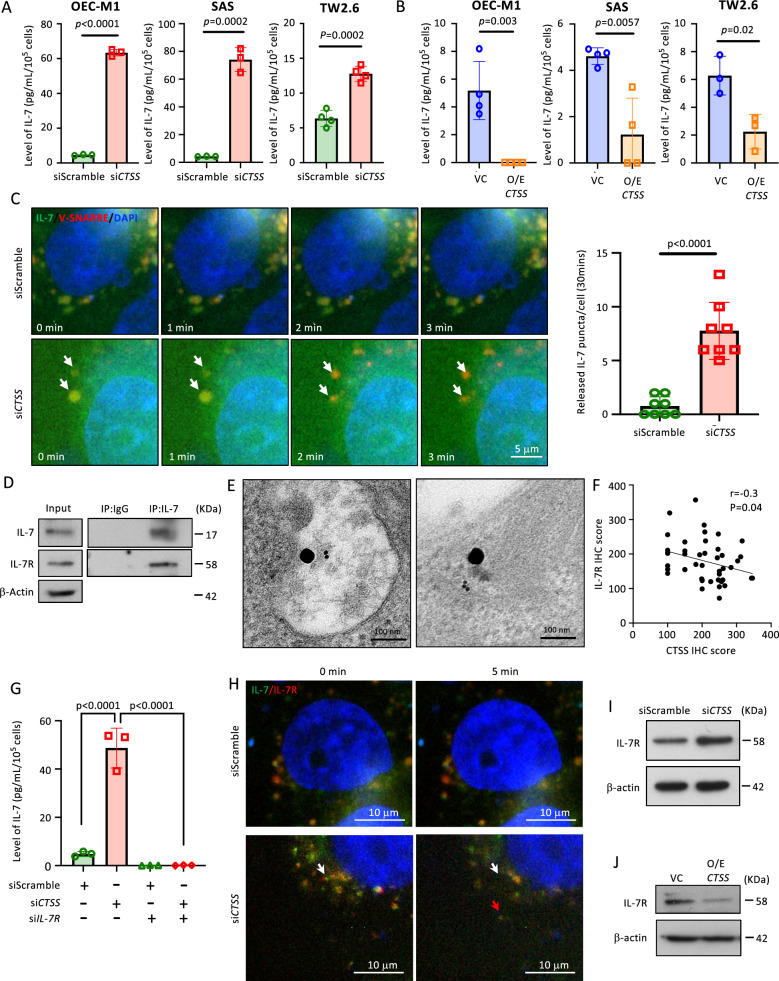
CTSS targets IL-7 receptor to inhibit IL-7 secretion. **A** & **B** The exocytotic levels of IL-7 were analyzed by the ELISA for (**A**) *CTSS*-silenced (si*CTSS*) or (**B**) *CTSS*-overexpressed (O/E *CTSS*) OEC-M1, SAS, and TW2.6 cells. Each dot represents one individual experiment. **C** Image snaps for the time-lapse immunofluorescent microscopy are shown here (Supplementary Video 1). Arrowheads indicates puncta with GFP signals that fad out over time. The bar graph shows quantification of the released IL-7 within 30 min. **D** IL-7 in OEC-M1 lysate was pulled down by immunoprecipitation (IP). The expression of IL-7 receptor (IL-7R) was then detected by Western blot (IB). **E** IL-7 and IL-7R in the si*CTSS*-transfected OEC-M1 cells were labeled with antibodies coupled with gold particles (40 nm and 10 nm, respectively). Their distribution was detected by transmission electron microscopy with the representative image shown in the panel. **F** The dot graph plot illustrates the correlation study between the IHC staining scores of CTSS and IL-7R in the in-house OC samples (N = 45). **G** The exocytotic levels of IL-7 in the culture medium of *CTSS*-silenced and/or *IL-7R*-silenced OEC-M1 cells were analyzed by the ELISA. **H** Snaps of time-lapse immunofluorescent microscopy (Supplementary video 2). White and red arrowheads indicated the location of the puncta at 0 and 5 min, respectively. **I** & **J** The expression of IL-7R in (**I**) *CTSS*-silenced or (**J**) -overexpressed OEC-M1 cells are analyzed by Western blot

OC cell lines (OEC-M1, SAS, and TW2.6) exhibited higher endogenous CTSS expression compared to the oral keratinocyte cell line (DOK). In these cells, CTSS knockdown markedly increased IL-7 secretion level in the culture medium **(**Fig. 3A and S5A, C, D**)**. In comparison, knockdown of cathepsin B (CTSB) or cathepsin L (CTSL) did not lead to significant changes in IL-7 secretion (Fig. S5E-F). Conversely, the IL-7 levels in CTSS-overexpressed cells were markedly suppressed (Fig. 3B and S5B, D). The changes in the fluorescent signal in IL-7-GFP- and V-SNARE-RFP (a granule vesicle surface marker)-expressed OEC-M1 cells were observed using time-lapse fluorescent microscopy to determine whether CTSS affects the intracellular transport and secretion of IL-7. GFP and RFP signals were co-localized in control cells. In contrast, CTSS-knockdown promoted the dissociation of GFP and RFP signals in the cells (Fig. 3C and Supplementary Video 1). These findings indicate that CTSS regulates IL-7 secretion.

Some cytokines depend on their paired receptors that have a high affinity for intracellular transport, such as IL-15/IL-15R [39]. We found that IL-7R co-precipitated with IL-7 in the cytoplasmic fraction, and that IL-7 co-localized with V-SNARE in the immunofluorescence microscopy results **(**Fig. 3D and S6A). Furthermore, immunogold labeling and TEM confirmed the co-localization of IL-7 and IL-7R in the intracellular vesicles (Fig. 3E). Together with a negative correlation that was found between the expression of CTSS and IL-7R in clinical tumor tissues (in-house samples and tissue array in Fig. 3F and S6B, respectively), these findings suggested that IL-7R participates in IL-7 transport. Next, the IL-7 levels significantly increased in si*CTSS*-transfected cells and were counteracted by the co-transfection of si*IL-7R* (Fig. 3G and S6C-D (another si*IL-7R*)**)**. Time-lapse microscopy showed the mobilization of IL-7-GFP/IL-7R-RFP puncta in CTSS-knockdown OEC-M1 cells but not in siScramble-transfected OEC-M1 cells (Fig. 3H and Supplementary Video 2), indicating that CTSS mediated the transport of IL-7/IL-7R granule vesicles. Additionally, western blot analysis revealed that the IL-7R level was consistently upregulated in the si*CTSS*-transfected cells (Fig. 3I and S6E) and downregulated in the CTSS-overexpressed cells (Fig. 3J and S6F). These findings indicate that IL-7R is a potential target of CTSS-regulated IL-7 transport.

### CTSS inhibits IL-7 secretion by interacting with the intracellular domain of IL-7R

**Fig. 4:**
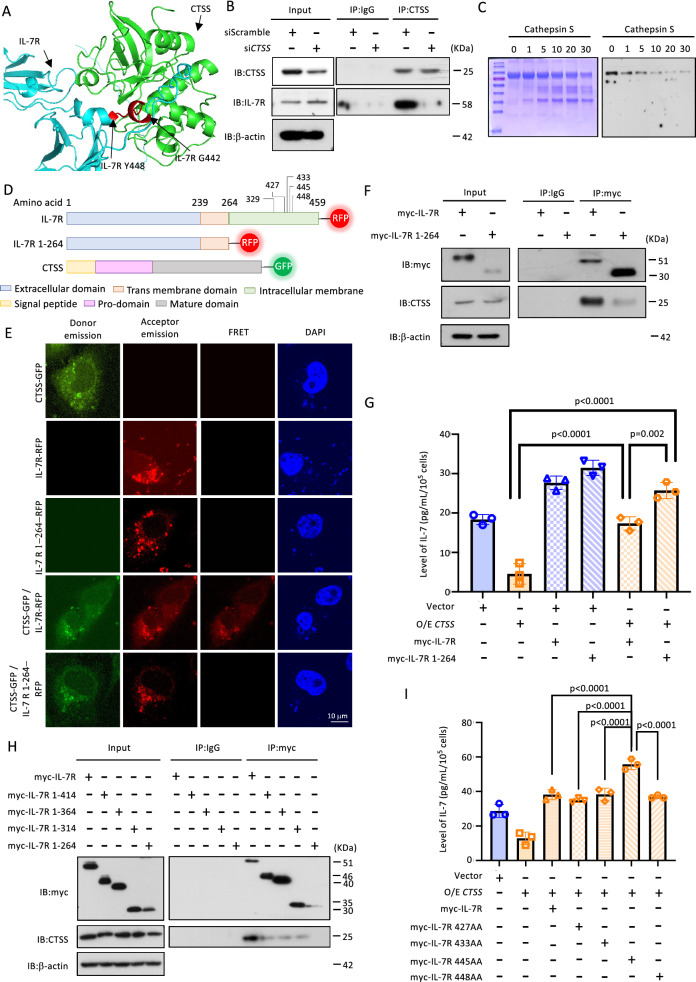
CTSS interacts with the intracellular domain of IL-7 receptor. **A** Schematic represent the protein–protein interaction of IL-7R (Cyan) and CTSS (green), which was predicted by the https://alphafoldserver.com. The interaction site of IL-7R is labeled in red. **B** OEC-M1 cells were pretreated with si*CTSS*. CTSS was pulled down by IP. The expression of IL-7R in the product was then detected by IB. **C** Recombinant IL-7R was incubated with CTSS for indicated duration. Samples were separated by the SDS-PAGE and applied to the Coomassie blue staining (left panel) and IB (right panel). **D** The schematic diagram shows DNA plasmid constructs of fluorescent-tagged IL-7R, truncated-form IL-7R (deletion of the amino acid sequence 264–459, IL-7R1-264), and CTSS. The cleavage sites predicted from https://platt.gatech.edu/PACMANS are indicated by connector lines. **E** Representative confocal microscopy image results of the FRET for the interaction of *CTSS-GFP* in the *IL-7R-RFP* or *IL-7R1-264-RFP* transfected OEC-M1 cells. Signals were obtained 48 h after transfection. **F** OEC-M1 cells were transfected with myc-tagged IL-7R or IL-7R1-264. Myc-tag was pulled down by IP. The expression of CTSS was then detected by IB. **G** The ELISA-detected IL-7 levels in the medium of *CTSS*-overexpressed OEC-M1 that was co-transfected with *IL-7R* or *IL-7R1-264* plasmids is shown in the dot-blot graph. **H** OEC-M1 cells were transfected with myc-tagged *IL-7R, IL-7R1-414*, *IL-7R1-364, IL-7R1-314, IL-7R1-264* plasmid. Myc-tag was pulled down by IP. The expression of CTSS was then detected by IB. **I** The ELISA-detected IL-7 levels in the medium of *CTSS*-overexpressed OEC-M1 that was co-transfected with myc-tagged *IL-7R, IL-7R 427AA, IL-7R 433AA, IL-7R 445AA, IL-7R 448AA* plasmid is shown in the dot-blot graph

The prediction of the CTSS-IL-7R protein–protein interaction revealed a potential interaction site (Fig. 4A). This was confirmed by immunoprecipitation, which revealed their interaction, and was further supported by the reduced pull-down IL-7R protein levels after CTSS-knockdown (Fig. 4B and S6G). Furthermore, the incubation of the recombinant CTSS protein with recombinant IL-7R protein demonstrated the proteolytic activity of CTSS (Fig. 4C).

Bioinformatics analysis revealed that a few amino acid residues were present in the intracellular domain of IL-7R, which could be recognized by CTSS for cleavage (Table 1). The interaction between CTSS and IL-7R was tracked using the FRET assay. For this purpose, a truncated form of IL-7R (IL-7R 1–264), deletion of the intracellular domain (amino acids 264–459) was synthesized (Fig. 4D). The results showed that the FRET RFP signal was detected in cells with IL-7R expression but not IL-7R 1–264 expression (Fig. 4E), indicating that the intracellular domain of IL-7R is the region for CTSS interaction. Additionally, a reduction in CTSS protein was observed in the myc-tagged pulldown assay in myc-IL-7R 1–264-transfected cells compared with myc-IL-7R-transfected cells (Fig. 4F and S7A). The amount of the secreted IL-7 was significantly reduced in CTSS-overexpressing cells. This effect was recovered by the overexpression of myc-IL-7R and, to a larger extent, by the overexpression of myc-IL-7R 1–264 (Fig. 4G and S7B). These findings indicate that the presence of the intracellular domain of IL-7R is critical for CTSS interaction. The precise site for the interaction was studied, with CTSS binding detected at a lower level by transfection of either myc-IL-7R 1–414, 1–364, 1–314, or 1–264 than in the intact form (Fig. 4H and S7C). The interaction site was suggested to be between 414 and 459. Eventually, in the retrieval study of CTSS-overexpressing cells, the interaction site was suggested to be at position 445 by detecting a high level of IL-7 when a mutation was introduced at that site (445/446) compared with other mutants or the intact myc-IL-7R (Fig. 4I and S7D).

**Table 1:**
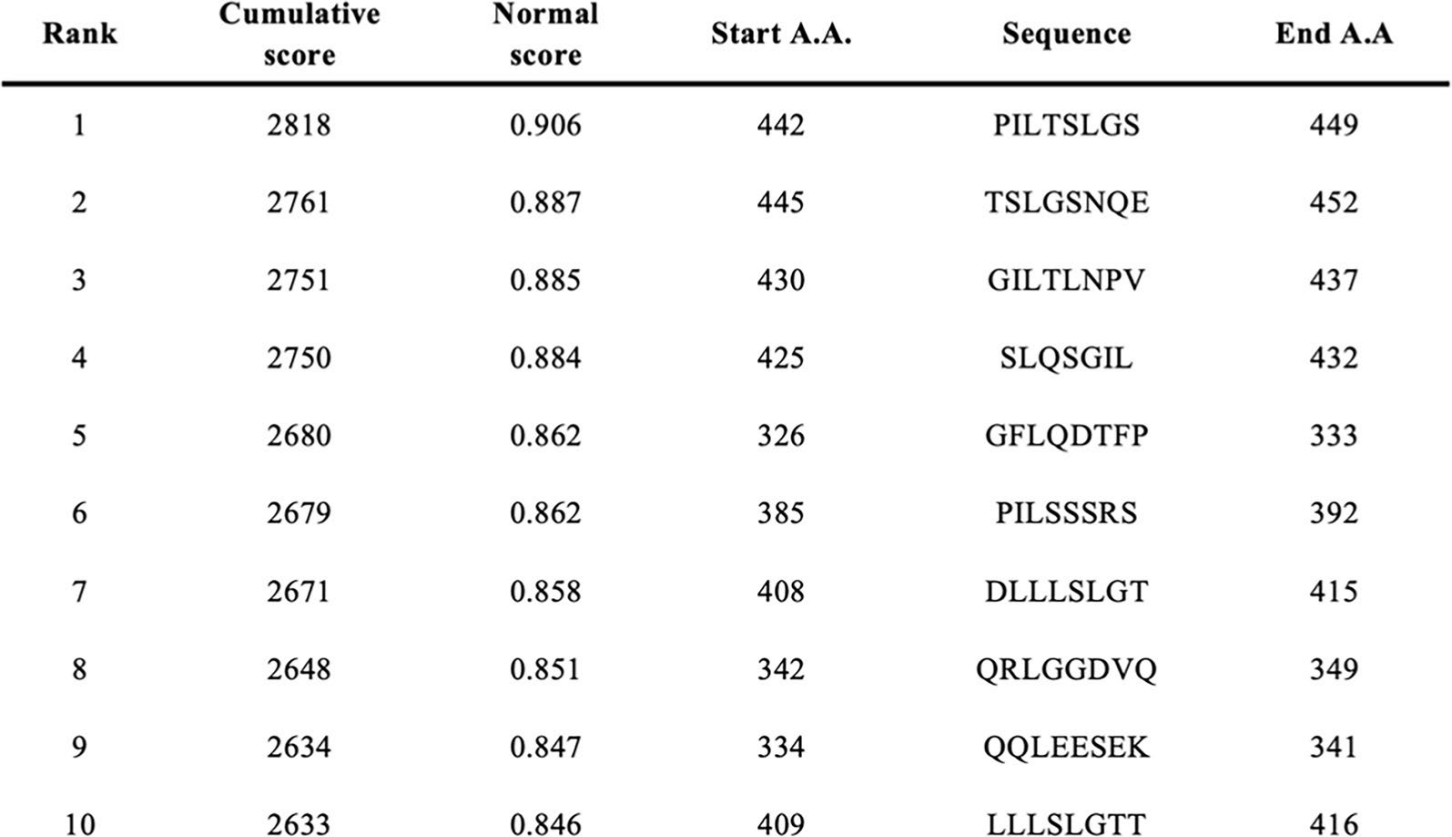
Predicted CTSS cleavage site of IL-7 receptor. The CTSS cleavage sites of IL-7R were predicted from https://platt.gatech.edu/PACMANS

### Targeting conventional trafficking and the mature form of CTSS enhances IL-7 secretion.

**Fig. 5:**
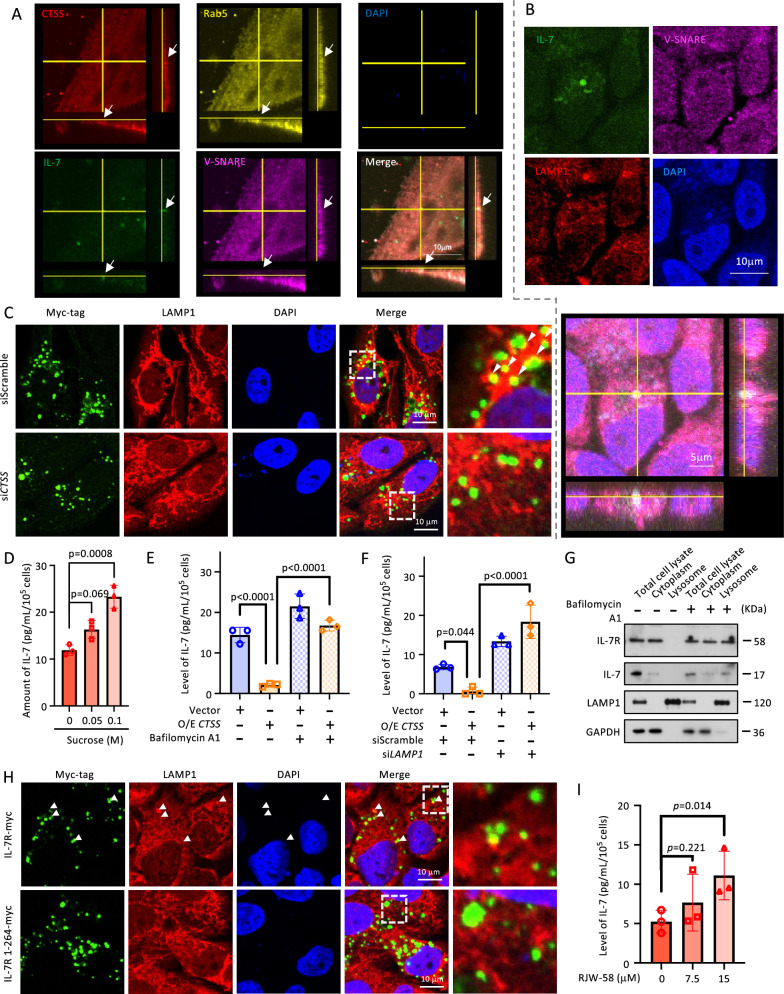
Inhibiting CTSS maturation upregulates IL-7 secretion. **A** Representative images of confocal microscopy for the IL-7-GFP-trasnfected OEC-M1 cells with V-SNARE, CTSS, and Rab5 labeled. **B** Representative images of confocal microscopy for the IL-7-GFP-trasnfected OEC-M1 cells with V-SNARE, and LAMP1 labeled. **C** Representative images of the confocal microscopy for the myc-tagged IL-7R, siScramble and si*CTSS*-co-transfected OEC-M1 cells. LAMP1 after 48 h transfection. **D** The exocytic IL-7 level in the medium of OEC-M1 that was co-incubated with sucrose for 72 h was measured. **E** & **F** The *CTSS*-overexpressed OEC-M1 cells were pretreated with (**E**) 3 nM BafA1 1 h prior, or were cotreated with (**F**) si*LAMP1*. The measured level of exocytic IL-7 in the medium is depicted in the graph. **G** OEC-M1 cells were treated with 3 nM BafA1 for 48 h. The protein expression level of IL-7R, IL-7, LAMP1, and GAPDH were detected by Western blotting analysis after lysosome isolation. **H** Representative images of the confocal microscopy for the myc-tagged IL-7R- or IL-7R1-264-transfected OEC-M1 cells. LAMP1 was labeled after 48-h transfection. (**I**) The level of exocytotic IL-7 from OEC-M1 that was co-incubated with RJW-58 for 72 h in a concentration-dependent manner (0.5- and onefold of IC_50_) was measured

Conventional trafficking through endosomes and lysosomes is essential for cathepsins to acquire proteolytic activity, known as the canonical route [40]. Results of immunofluorescence microscopy revealed the co-localization of IL-7, V-SNARE, CTSS, and Rab5 (an early endosome marker) in OEC-M1 cells (Fig. 5A and S8A), indicating the fusion of IL-7-containing granule vesicles with CTSS-containing early endosomes. These IL-7 vesicles were found to co-localize with lysosomes, as indicated by the co-localization of IL-7, V-SNARE, and the lysosome marker LAMP1 **(**Fig. 5B and S8B). We further demonstrated that the colocalization of IL-7R and LAMP1 was decreased in the CTSS-knockdown OC cells **(**Fig. 5C**)**.

Functionally, sucrose, an endosomal rupture, enhanced IL-7 secretion** (**Fig. 5D and S9A) [41], indicating that the endosomal process has a crucial role in mediating the intracellular level of CTSS. Similarly, lysosomal inhibition by BafA1 or si*LAMP1* counteracted the decreased IL-7R expression and IL-7 secretion induced by CTSS overexpression **(**Fig. 5E–F and S9B-D**)**. The expression of IL-7R and IL-7 in lysosomes increased upon treatment with BafA1, indicating reduced degradation (Fig. 5G). Importantly, LAMP1 co-localized with IL-7R in IL-7R-overexpressing cells but not IL-7R 1–264-overexpressing cells (Fig. 5H). These findings indicate that the canonical route of CTSS is responsible for interfering with the transport of IL-7-containing granule vesicles.

Given the pivotal role of the CTSS, RJW-58 was used to target mature CTSS. Consistent with the knockdown studies, RJW-58 decreased CTSS protease activity and increased IL-7 secretion in a concentration-dependent manner (Fig. 5I and S9E-F**)**, whereas inhibitors of cathepsin B, cathepsin L, or pan-cathepsins did not demonstrate the same effect (Fig. S9G-H). In the NHRI-HN1-bearing mouse model, a 5-day RJW-58 was administered in accordance with the optimal schedule for CTSS delivery [26]. The result showed a significant anti-tumor effect of RJW-58, which could be counteracted by co-administration of the αIL-7 without causing a change in the mouse body weight (Fig. S10A–D). The results of the IHC consistently showed lower CTSS expression, higher IL-7 expression, and more infiltration of CD8^+^ T cells in tumor tissue samples from the treatment groups than in the control group (Fig. S10E). FACS analysis revealed no significant changes in CD3^+^ and CD4^+^ T cells (Fig. S10F-G). A significantly higher CD8^+^/CD3^+^ ratio was observed in the treatment group in response to co-treatment with the αIL-7 was similarly observed (Fig. S10H). These CD8^+^ T cells showed characteristics of decreased PD-1^+^ expression but not granzyme B^+^ expression (Fig. S10H). These findings indicate that RJW-58 effectively modulates tumor immunity in a compatible manner.

### RJW-58 exhibits an immunomodulatory effect and enhances the anti-cancer effects of the αPD-1 in OC mouse models

**Fig. 6:**
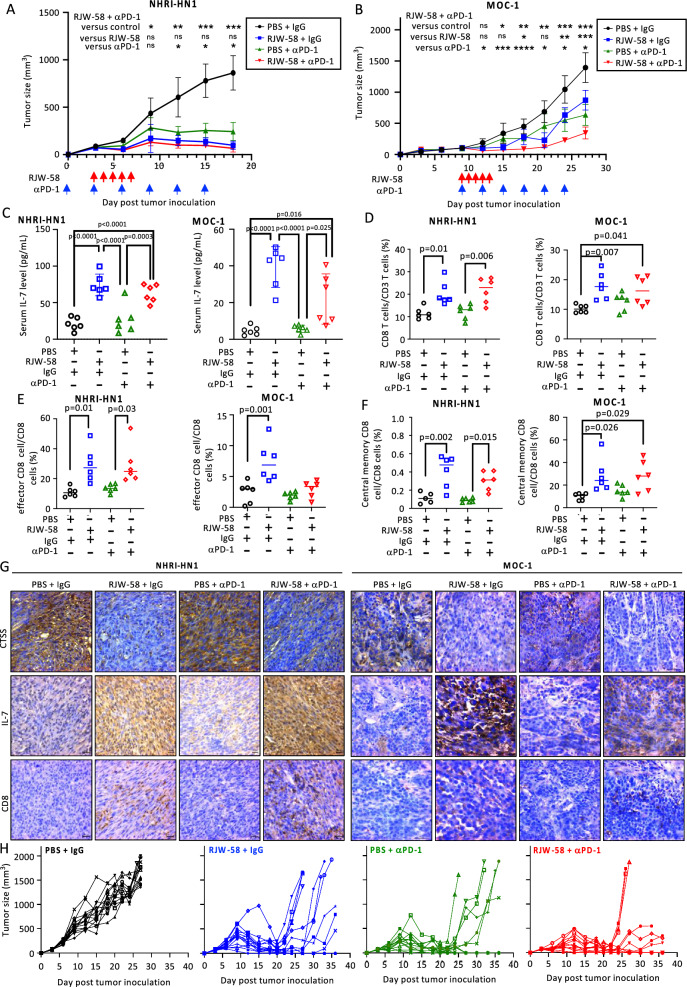
Enhanced anti-tumor effect by combining RJW-58 and the αPD-1 in the OC syngeneic mouse model. **A**&**B** The tumor volume of the subcutaneously-inoculated (**A**) NHRI-HN1 cells or (**B**) MOC-1 cells is plotted. Mice were grouped by treatment with RJW-58, αPD-1 or combination (N = 6 for each group). Red and blue arrowheads indicate days for RJW-58 and antibody administration, respectively. **C** The serum of each mouse was collected on day 18 and day 27, before the end of the study. The level of IL-7 was measured by ELISA subsequently. **D**–**G** NHRI-HN1- or MOC-1 bearing mice were sacrificed on day 18 and day 27, respectively. Tumors were extracted for (**D**-**F**) FACS analysis (with the percentage of (**D**) CD8^+^ T cells in CD45^+^ cells, (**E**) effector CD8^+^ T cells in CD8^+^ cells, and (**F**) CD8 Tcm in CD8^+^ T cells of each mouse shows in the dot graph), and for (**G**) IHC staining (with the representative photos of CTSS, IL-7, and CD8 shown in the panels). **H** A longer-term observation of NHRI-HN1-carrying mice that had followed the identical treatment course indicated in Fig. 6A. They were then kept until humane endpoints or until day 36. The tumor volume of each mouse is plotted as a curve in the graph according to the treatment received. Each treatment group included 14 mice in the beginning. There were non-relevant deaths of one mouse in both PBS + αPD-1 and RJW-58 + αPD-1 groups on day 15 and day 12, respectively, and thus were excluded from the result

The combination effect of RJW-58 with the αPD-1 was investigated in vivo. Given that tumor growth was entirely suppressed in sh*Ctss*-NHRI-HN1 tumor cells in a long-term manner (Fig. S11), an administration schedule with short-term RJW-58 was used to avoid outperformance over the effect of the αPD-1. All treatments inhibited tumor growth in NHRI-HN1- and MOC-1-bearing mice, regardless of early or late treatment after tumor inoculation, without causing a change in the mouse body weight (Fig. 6A, B and S12A-B). The growth rate resumed in MOC-1 tumors shortly after the termination of RJW-58 but remained slower in the combination group than in monotherapies (Fig. 6B). Conversely, in NHRI-HN1-bearing mice, all treatments effectively suppressed tumor growth compared to the control group; however, no statistically significant differences were observed among the treatment groups. (Fig. 6A). The molecular studies of both tumors were compatible with the in vitro studies, with enhanced serum IL-7 levels and increased CD8^+^ T cells by RJW-58 treatment (Fig. 6C–D). The characteristics of these CD8^+^ T cells showed an increase in the effector CD8^+^ T cell subpopulation (Fig. 6E) and repopulation of memory CD8^+^ T cells skewing toward the Tcm subsets in the RJW-58-treated tumor group (Fig. 6F). No differences were observed in the CD4^+^ T cell subsets, granzyme B^+^/CD8^+^ cells, PD-1^+^/CD8^+^ cells, CD8 Treg cells, naïve CD8^+^ T cells, CD8 Tem cells, and CD8 Trm cells (Fig. S12C-D and S13). This finding was supported by IHC, which showed that RJW-58 with or without the αPD-1 caused reduced CTSS expression, increased IL-7 expression, and increased CD8^+^-T cell infiltration in the tumor tissues (Fig. 6G and S14). Given the consistent skewing toward the Tcm in the memory CD8^+^ T cells (Fig. 6F), an extra treatment-free observation period was applied after the completion of the drug administration schedule in the NHRI-HN1 mouse model. The results showed that more mice had tumors that regrew in the RJW-58 and αPD-1 groups compared with the combination group (tumor size > 1000 mm^3^: 7/14 (50%) vs. 6/13 (46%) vs. 3/13 (23%), respectively) (Fig. 6H). These findings indicated that RJW-58 could promote the anti-cancer effect of the αPD-1 and deliver various advantages over the use of monotherapy.

## Discussion

The cathepsin family is well known to regulate multiple proteins within cytoplasmic membrane-bound vesicles [42]. The findings indicate that CTSS targets IL-7R by recognizing the intracellular domain to block IL-7 intracellular transport (Fig. 4). Additionally, the findings indicated that the early endosome plays a major role in this regulation, which determines the destination of fusion vesicles by transporting them to the lysosome for degradation or recycling to the cell membrane for secretion [43]. This canonical functioning route of CTSS plays an active role in mediating intracellular proteins, such as the regulation of endosomal epidermal growth factor receptors [44]. However, the reduced IL-7 secretion by CTSS overexpression could not be completely counteracted when replacing myc-IL-7R with myc-IL-7R 1–264 (Fig. 4G). The non-canonical functioning routes of CTSS that occurred in the extracellular space or nucleus could be responsible. For example, the activation of IL-36γ by the extracellular and intracellular CTSS in epithelial tissue contributes to psoriatic inflammation [21]. The cleavage study conducted in a neutral environment showed fewer limitations on the pH range for CTSS functioning (Fig. 4C), highlighting one of its unique features [45]. These findings made it difficult to rule out other factors and mechanisms involved in CTSS-inhibited IL-7 secretion. However, this study still showed significant rescue of IL-7 levels by sucrose against endosomes and BafA1/si*LAMP1* against lysosomes (Fig. 5C–E), highlighting the canonical route that prompted CTSS to gain endopeptidase function.

Multiple routes for cytokine secretion from cells have been identified. Structurally, IL-7 has classical signal peptides that contain a glycosylation sequence as a hallmark for translocation across the endoplasmic reticulum (ER) and Golgi apparatus [46, 47]. The driving mechanism for this type of transport remains unclear. We identified a novel mechanism by which intracellular IL-7R accompanies IL-7. We further demonstrated that the IL-7-IL7R complex colocalized with Calnexin, GOLGA5, and E-cadherin indicating that IL-7 secretion may work through the ER-Golgi system (Fig. S15). These cytokine receptors are anticipated to play unconventional roles when located in cells, and some of them participate in multiple trafficking processes of their paired cytokines. The IL-4Rα subunit mediates the recruitment of IL-4 from eosinophilic granules to secretory vesicles [48]. Furthermore, the IL-15-IL-15R complex is pre-assembled in the ER and trafficked through the secretory pathway [39]. It was thus not surprising that IL-7R was identified as a critical factor that promotes IL-7 intracellular transport and secretion **(**Fig. 3G**)**. Supportively, the common gamma chain (IL-2Rγ), which forms a heterodimer with IL-7R and interacts with IL-7 [49], was recognized in the pull-down IL-7 complex (Fig. S16). IL-7R is conventionally expressed in lymphocytes and detected in tumor cells, including lung and breast cancer [50]. These findings indicate that IL-7R is potentially responsible for packing IL-7 into granule vesicles for secretion in OC cells.

The results showed that the primary anti-tumor mechanism for CTSS suppression operated through immunomodulation in our mouse model (Fig. 1, 2, and 6). In several inconclusive clinical trials, CTSS inhibitors were reported to have immunomodulatory effects [51, 52]. The lower toxicity observed in these studies, as supported by the lack of growth defects in CTSS-knockout mice [26], indicates limited damage to target or normal cells. Furthermore, the results showed that systemic depletion could be misinterpreted as the local impact of the tumor cytokine. Therefore, the significant difference in T cell proliferation observed in the ex vivo study, by manipulating the CM of the CTSS-knockdown tumor with or without the IL-7 recombinant protein, pursued the interaction that occurred in the local tissue (Fig. 2G–H). A consistently negative correlation between CTSS expression and CD8^+^ T-cell infiltration in the samples, especially T1 and T2 classifications with less influence from extensive microenvironmental components comparing to the larger tumors (Fig. 1G) [53], further underscores its clinical relevance. However, besides cytokine regulation, CTSS affects immunity through multiple routes, including converting MHC classes [54]. CTSS inhibition caused antigen diversification in non-Hodgkin lymphoma that switched immunity by promoting CD8^+^ T cell polyclonal expansion and suppressing CD4^+^ T cells [55]. In contrast, our results showed a narrower impact on CD8^+^ T cells but not on CD4^+^ counterparts (Fig. 2D, 6D, S4, and S12C-D). Although the involvement of other lymphocytes or factors could not be excluded, the primary reaction for CD8^+^ T cells accounted for the anti-tumor effect in local immunity. Therefore, the trend of the memory subsets related to IL-7, including a tendency skewing toward Tcm, was crucial (Fig. 2F and 6F). Additionally, the results showed inconsistent or unaltered findings in the effector and Treg subsets of CD8^+^ T cells and PD-1 and granzyme B expression (Fig. S4). Given that IL-7R is a critical factor for memory T cells, the significance of IL-7 should not be overlooked [56]. These findings indicate that CTSS negatively regulates IL-7. This negative regulation can facilitate the maintenance of long-lasting memory cells, thereby enhancing their plasticity and systemic distribution [57–59].

Although IL-7 is crucial for lymphocyte maintenance, a sarcoma clinical trial showed that adjuvant IL-7 increased T cell infiltration without providing anti-cancer benefits [60]. Herein, syngeneic mouse models using different CTSS-targeting strategies yielded contrasting results (Fig. 6), which could be attributed to variations in immune properties across tumor types. To the best of our knowledge, no clinical trials have investigated CTSS inhibitors in oncology. RJW-58 had higher CTSS-target specificity than other inhibitors [24]; therefore, served as a good candidate for evaluation for the immunotherapy impact. Herein, the combination of RJW-58 and the αPD-1 led to a more persistent therapeutic advantage in the mouse model, with memory CD8^+^ T cells skewing toward the Tcm feature (Fig. 6F). Supportively, a higher Tcm level indicates a rapid proliferative response of T cells to antigens [61], which was associated with better ICT response rates and longer disease-free survival in lung cancer [62]. This advantage stems from properties that differ from those of other cytokines that directly stimulate lymphocytes activation. Notably, IL-7 antagonizes multiple inhibitory networks when administered with immunotherapies as an adjunct treatment [63]. Moreover, CTSS suppression is expected to enhance ICT through other mechanisms involving different lymphocytes [55]. Although cancer and normal cells exhibit differential CTSS expression levels (Fig. S5C), the compound lacks specificity for malignant cells. This limitation raises concerns about potential off-target effects, which could be mitigated through further formulation optimization, such as nanoparticle package and antibody conjugation [64, 65]. Nonetheless, our study employing this specific CTSS-targeting strategy, cyto-immunotherapy, showed strong evidence by enhancing current ICT strategies for OC management.

## Conclusions

This study showed the regulatory role of CTSS in the secretion of IL-7 mediated by the recognition of IL-7R during intracellular transport from a molecular perspective. Additionally, the results showed that CTSS inhibition enhanced anti-tumor immunity associated with CD8^+^ T cells in vivo and in vitro. Therefore, investigating its translational value based on our results is crucial.

## Supplementary Information

Supplementary file 1.

## Data Availability

Data supporting the findings of this study are available from the corresponding authors Jang-Yang Chang and Kwang-Yu Chang on request.

## Abbreviations

4-NQO: 4-Ntitroquinoline 1-oxide
αCD8: Anti CD8b antibody
αIL-7: Anti IL-7 antibody
αPD-1: Anti PD-1 antibody
BafA1: Bafilomycin A1
CD4^+/^CD8^+^ Treg: CD4^+/^CD8^+^ regulatory T cell
CTSB: Cathepsin B
CTSL: Cathepsin L
CTSS: Cathepsin S
CM: Conditioned medium
ELISA: Enzyme-linked immunosorbent assay
FACS: Fluorescence activated cell sorting
FRET: Fluorescence resonance energy transfer
HNC: Head and neck cancer
ICT: Immune checkpoint therapy
IHC: Immunohistochemistry
IL-2: Interleukin-2
IL-7: Interleukin-7
IL-7R: IL-7 receptor
IP: Immunoprecipitation assay
MHC: Major histocompatibility complex
OC: Oral cancer
Tcm: Central memory subsets of T cells
Tem: Effector memory subsets of T cells
TEM: Transmission electron microscopy
Trm: Tissue-resident memory subsets of T cells
WST: Water-soluble tetrazolium

## References

[1] Bray F, Ferlay J, Soerjomataram I, Siegel RL, Torre LA, Jemal A. Global cancer statistics 2018: GLOBOCAN estimates of incidence and mortality worldwide for 36 cancers in 185 countries. CA Cancer J Clin. 2018;68(6):394–424. 30207593 10.3322/caac.21492

[2] De Angelis R, Sant M, Coleman MP, Francisci S, Baili P, Pierannunzio D, et al. Cancer survival in Europe 1999–2007 by country and age: results of EUROCARE–5-a population-based study. Lancet Oncol. 2014;15(1):23–34. 24314615 10.1016/S1470-2045(13)70546-1

[3] Farlow JL, Brenner JC, Lei YL, Chinn SB. Immune deserts in head and neck squamous cell carcinoma: a review of challenges and opportunities for modulating the tumor immune microenvironment. Oral Oncol. 2021;120: 105420. 34218062 10.1016/j.oraloncology.2021.105420 PMC8753751

[4] Berraondo P, Sanmamed MF, Ochoa MC, Etxeberria I, Aznar MA, Perez-Gracia JL, et al. Cytokines in clinical cancer immunotherapy. Br J Cancer. 2019;120(1):6–15. 30413827 10.1038/s41416-018-0328-y PMC6325155

[5] Bradley LM, Haynes L, Swain SL. IL-7: maintaining T-cell memory and achieving homeostasis. Trends Immunol. 2005;26(3):172–6. 15745860 10.1016/j.it.2005.01.004

[6] Barata JT, Durum SK, Seddon B. Flip the coin: IL-7 and IL-7R in health and disease. Nat Immunol. 2019;20(12):1584–93. 31745336 10.1038/s41590-019-0479-x

[7] Mackall CL, Fry TJ, Gress RE. Harnessing the biology of IL-7 for therapeutic application. Nat Rev Immunol. 2011;11(5):330–42. 21508983 10.1038/nri2970 PMC7351348

[8] Andersson A, Yang SC, Huang M, Zhu L, Kar UK, Batra RK, et al. IL-7 promotes CXCR3 ligand-dependent T cell antitumor reactivity in lung cancer. J Immunol. 2009;182(11):6951–8. 19454692 10.4049/jimmunol.0803340

[9] Sportes C, Babb RR, Krumlauf MC, Hakim FT, Steinberg SM, Chow CK, et al. Phase I study of recombinant human interleukin-7 administration in subjects with refractory malignancy. Clin Cancer Res. 2010;16(2):727–35. 20068111 10.1158/1078-0432.CCR-09-1303 PMC2808195

[10] vonFreedenJeffry U, Solvason N, Howard M, Murray R. The earliest T lineage-committed cells depend on IL-7 for Bcl-2 expression and normal cell cycle progression. Immunity. 1997;7(1):147–54. 9252127 10.1016/s1074-7613(00)80517-8

[11] Judge CJ, Kostadinova L, Sherman KE, Butt AA, Falck-Ytter Y, Funderburg NT, et al. CD56 NK IL-7Rα expression negatively associates with HCV level, and IL-7-induced NK function is impaired during HCV and HIV infections. J Leukocyte Biol. 2017; 102(1):171-84 10.1189/jlb.5A1116-456R PMC5470838 28400540

[12] Chen D, Tang TX, Deng H, Yang XP, Tang ZH. Interleukin-7 biology and its effects on immune cells: mediator of generation, differentiation, survival, and homeostasis. Front Immunol. 2021;12:1. 10.3389/fimmu.2021.747324 PMC8674869 34925323

[13] Perales MA, Goldberg JD, Yuan JD, Koehne G, Lechner L, Papadopoulos EB, et al. Recombinant human interleukin-7 (CYT107) promotes T-cell recovery after allogeneic stem cell transplantation. Blood. 2012;120(24):4882–91. 23012326 10.1182/blood-2012-06-437236 PMC3520625

[14] Shourian M, Beltra JC, Bourdin B, Decaluwe H. Common gamma chain cytokines and CD8 T cells in cancer. Semin Immunol. 2019;42: 101307. 31604532 10.1016/j.smim.2019.101307

[15] Di Nitto C, Ravazza D, Gilardoni E, Look T, Sun M, Prodi E, et al. An IL-7 fusion protein targeting EDA fibronectin upregulates TCF1 on CD8+ T-cells, preferentially accumulates to neoplastic lesions, and boosts PD-1 blockade. J Immunother Cancer. 2024;12(8): e008504. 39142716 10.1136/jitc-2023-008504 PMC11332014

[16] Olson OC, Joyce JA. Cysteine cathepsin proteases: regulators of cancer progression and therapeutic response. Nat Rev Cancer. 2015;15(12):712–29. 26597527 10.1038/nrc4027

[17] McDowell SH, Gallaher SA, Burden RE, Scott CJ. Leading the invasion: the role of Cathepsin S in the tumour microenvironment. Biochim Biophys Acta Mol Cell Res. 2020;1867(10): 118781. 32544418 10.1016/j.bbamcr.2020.118781

[18] Wilkinson RD, Williams R, Scott CJ, Burden RE. Cathepsin S: therapeutic, diagnostic, and prognostic potential. Biol Chem. 2015;396(8):867–82. 25872877 10.1515/hsz-2015-0114

[19] Wang B, Sun J, Kitamoto S, Yang M, Grubb A, Chapman HA, et al. Cathepsin S controls angiogenesis and tumor growth via matrix-derived angiogenic factors. J Biol Chem. 2006;281(9):6020–9. 16365041 10.1074/jbc.M509134200

[20] Hsing LC, Rudensky AY. The lysosomal cysteine proteases in MHC class II antigen presentation. Immunol Rev. 2005;207:229–41. 16181340 10.1111/j.0105-2896.2005.00310.x

[21] Ainscough JS, Macleod T, McGonagle D, Brakefield R, Baron JM, Alase A, et al. Cathepsin S is the major activator of the psoriasis-associated proinflammatory cytokine IL-36gamma. Proc Natl Acad Sci U S A. 2017;114(13):E2748–57. 28289191 10.1073/pnas.1620954114 PMC5380102

[22] Burden RE, Gormley JA, Kuehn D, Ward C, Kwok HF, Gazdoiu M, et al. Inhibition of Cathepsin S by Fsn0503 enhances the efficacy of chemotherapy in colorectal carcinomas. Biochimie. 2012;94(2):487–93. 21896304 10.1016/j.biochi.2011.08.017

[23] Seo SU, Min KJ, Woo SM, Kwon TK. Z-FL-COCHO, a cathepsin S inhibitor, enhances oxaliplatin-mediated apoptosis through the induction of endoplasmic reticulum stress. Exp Mol Med. 2018;50(8):1–11. 30120227 10.1038/s12276-018-0138-6 PMC6098103

[24] Lin HH, Chen SJ, Shen MR, Huang YT, Hsieh HP, Lin SY, et al. Lysosomal cysteine protease cathepsin S is involved in cancer cell motility by regulating store-operated Ca2+ entry. Biochim Biophys Acta Mol Cell Res. 2019;1866(12): 118517. 31340164 10.1016/j.bbamcr.2019.07.012

[25] Chen JC, Uang BJ, Lyu PC, Chang JY, Liu KJ, Kuo CC, et al. Design and synthesis of alpha-ketoamides as cathepsin S inhibitors with potential applications against tumor invasion and angiogenesis. J Med Chem. 2010;53(11):4545–9. 20481438 10.1021/jm100089e

[26] Chen SJ, Chen LH, Yeh YM, Lin CK, Lin PC, Huang HW, et al. Targeting lysosomal cysteine protease cathepsin S reveals immunomodulatory therapeutic strategy for oxaliplatin-induced peripheral neuropathy. Theranostics. 2021;11(10):4672–87. 33754020 10.7150/thno.54793 PMC7978314

[27] Tangeda V, Lo YK, Babuharisankar AP, Chou HY, Kuo CL, Kao YH, et al. Lon upregulation contributes to cisplatin resistance by triggering NCLX-mediated mitochondrial Ca(2+) release in cancer cells. Cell Death Dis. 2022;13(3):241. 35296653 10.1038/s41419-022-04668-1 PMC8927349

[28] Hsiao SY, Weng SM, Hsiao JR, Wu YY, Wu JE, Tung CH, et al. MiR-455-5p suppresses PDZK1IP1 to promote the motility of oral squamous cell carcinoma and accelerate clinical cancer invasion by regulating partial epithelial-to-mesenchymal transition. J Exp Clin Cancer Res. 2023;42(1):40. 36737832 10.1186/s13046-023-02597-1 PMC9896797

[29] Chen YL, Yen YC, Jang CW, Wang SH, Huang HT, Chen CH, et al. Ephrin A4-ephrin receptor A10 signaling promotes cell migration and spheroid formation by upregulating NANOG expression in oral squamous cell carcinoma cells. Sci Rep. 2021;11(1):644. 33436772 10.1038/s41598-020-80060-3 PMC7804096

[30] Yen YC, Shiah SG, Chu HC, Hsu YM, Hsiao JR, Chang JY, et al. Reciprocal regulation of microRNA-99a and insulin-like growth factor I receptor signaling in oral squamous cell carcinoma cells. Mol Cancer. 2014;13:6. 24410957 10.1186/1476-4598-13-6 PMC3895693

[31] Chen YL, Liu KJ, Jang CW, Hsu CC, Yen YC, Liu YL, et al. ERK activation modulates cancer stemness and motility of a novel mouse oral squamous cell carcinoma cell line. Cancers (Basel). 2019;12(1):61. 31878324 10.3390/cancers12010061 PMC7016611

[32] Sheu MJ, Chou CL, Yang CC, Lee SW, Tian YF, Lin CY, et al. Low BRCA2 expression predicts poor prognoses in patients with rectal cancer receiving chemoradiotherapy. Pathol Res Pract. 2020;216(5): 152922. 32249003 10.1016/j.prp.2020.152922

[33] Schollbach J, Kircher S, Wiegering A, Seyfried F, Klein I, Rosenwald A, et al. Prognostic value of tumour-infiltrating CD8+ lymphocytes in rectal cancer after neoadjuvant chemoradiation: is indoleamine-2,3-dioxygenase (IDO1) a friend or foe? Cancer Immunol Immunother. 2019;68(4):563–75. 30671614 10.1007/s00262-019-02306-y PMC11028246

[34] Lin Y-S, Tsai Y-C, Li C-J, Wei T-T, Wang J-L, Lin B-W, Wu Y-N, Wu S-R, Lin S-C, Lin S-C. Overexpression of NUDT16L1 sustains proper function of mitochondria and leads to ferroptosis insensitivity in colorectal cancer. Redox Biology 2024;77:103358. 10.1016/j.redox.2024.103358 39317106 10.1016/j.redox.2024.103358 PMC11465047

[35] Hunt KS, Alspach E. Battle within the sexes: differences in male and female immunity and the impact on antitumor responses. Cancer Immunol Res. 2024;12(1):17–25. 37939008 10.1158/2326-6066.CIR-23-0005

[36] Golubovskaya V, Wu LJ. Different subsets of T cells, memory, effector functions, and CAR-T immunotherapy. Cancers. 2016;8(3):36. 26999211 10.3390/cancers8030036 PMC4810120

[37] Herndler-Brandstetter D, Schwaiger S, Veel E, Fehrer C, Cioca DP, Almanzar G, et al. CD25-expressing CD8+ T cells are potent memory cells in old age. J Immunol. 2005;175(3):1566–74. 16034095 10.4049/jimmunol.175.3.1566

[38] Yu SF, Lao SH, Yang BY, Wu CY. Tissue-resident memory-like CD8 T cells exhibit heterogeneous characteristics in tuberculous pleural effusion. J Immunol Res. 2021;1:6643808. 10.1155/2021/6643808 PMC8084674 33977110

[39] Duitman EH, Orinska Z, Bulanova E, Paus R, Bulfone-Paus S. How a cytokine is chaperoned through the secretory pathway by complexing with its own receptor: lessons from interleukin-15 (IL-15)/IL-15 receptor alpha. Mol Cell Biol. 2008;28(15):4851–61. 18505820 10.1128/MCB.02178-07 PMC2493373

[40] Brix K. Host cell proteases: cathepsins. In: Böttcher-Friebertshäuser E, Garten W, Klenk H, editors. Springer. Cham; 2018.

[41] Cervia LD, Chang CC, Wang L, Yuan F. Distinct effects of endosomal escape and inhibition of endosomal trafficking on gene delivery via electrotransfection. PLoS ONE. 2017;12(2): e0171699. 28182739 10.1371/journal.pone.0171699 PMC5300164

[42] Anes E, Pires D, Mandal M, Azevedo-Pereira JM. Spatial localization of cathepsins: implications in immune activation and resolution during infections. Front Immunol. 2022;13: 955407. 35990632 10.3389/fimmu.2022.955407 PMC9382241

[43] Omari S, Roded A, Eisenberg M, Ali H, Fukuda M, Galli SJ, et al. Mast cell secretory granule fusion with amphisomes coordinates their homotypic fusion and release of exosomes. Cell Rep. 2024;43(7): 114482. 38985670 10.1016/j.celrep.2024.114482

[44] Huang CC, Lee CC, Lin HH, Chang JY. Cathepsin S attenuates endosomal EGFR signalling: a mechanical rationale for the combination of cathepsin S and EGFR tyrosine kinase inhibitors. Sci Rep. 2016;6:29256. 27387133 10.1038/srep29256 PMC4937378

[45] Kirschke H, Wiederanders B, Bromme D, Rinne A, Cathepsin S. from bovine spleen purification, distribution, intracellular localization and action on proteins. Biochem J. 1989;264(2):467–73. 2690828 10.1042/bj2640467 PMC1133603

[46] Park JJ, Loh YP. How peptide hormone vesicles are transported to the secretion site for exocytosis. Mol Endocrinol. 2008;22(12):2583–95. 18669645 10.1210/me.2008-0209 PMC2626200

[47] Duitman EH, Orinska Z, Bulfone-Paus S. Mechanisms of cytokine secretion: a portfolio of distinct pathways allows flexibility in cytokine activity. Eur J Cell Biol. 2011;90(6–7):476–83. 21439673 10.1016/j.ejcb.2011.01.010

[48] Spencer LA, Melo RC, Perez SA, Bafford SP, Dvorak AM, Weller PF. Cytokine receptor-mediated trafficking of preformed IL-4 in eosinophils identifies an innate immune mechanism of cytokine secretion. Proc Natl Acad Sci U S A. 2006;103(9):3333–8. 16492782 10.1073/pnas.0508946103 PMC1413889

[49] Wang C, Kong L, Kim S, Lee S, Oh S, Jo S, et al. The role of IL-7 and IL-7R in cancer pathophysiology and immunotherapy. Int J Mol Sci. 2022;23(18):10412. 36142322 10.3390/ijms231810412 PMC9499417

[50] Suzuki K, Kadota K, Sima CS, Nitadori J, Rusch VW, Travis WD, et al. Clinical impact of immune microenvironment in stage I lung adenocarcinoma: tumor interleukin-12 receptor beta2 (IL-12Rbeta2), IL-7R, and stromal FoxP3/CD3 ratio are independent predictors of recurrence. J Clin Oncol. 2013;31(4):490–8. 23269987 10.1200/JCO.2012.45.2052 PMC3731922

[51] Gadola SD, Farber P, Posch MG, Nagel S, Canducci F. An open-label phase 2a study investigating the efficacy and safety of a cathepsin S inhibitor in patients with moderate-to-severe psoriasis. J Am Acad Dermatol. 2022;87(5):1089–91. 34906661 10.1016/j.jaad.2021.09.074

[52] Bentley D, Fisher BA, Barone F, Kolb FA, Attley G. A randomized, double-blind, placebo-controlled, parallel group study on the effects of a cathepsin S inhibitor in primary Sjogren’s syndrome. Rheumatology (Oxford). 2023;62(11):3644–53. 36864622 10.1093/rheumatology/kead092 PMC10629789

[53] Liu ZL, Meng XY, Bao RJ, Shen MY, Sun JJ, Chen WD, et al. Single cell deciphering of progression trajectories of the tumor ecosystem in head and neck cancer. Nat Commun. 2024;15(1):2595. 38519500 10.1038/s41467-024-46912-6 PMC10959966

[54] Bania J, Gatti E, Lelouard H, David A, Cappello F, Weber E, et al. Human cathepsin S, but not cathepsin L, degrades efficiently MHC class II-associated invariant chain in nonprofessional APCs. Proc Natl Acad Sci U S A. 2003;100(11):6664–9. 12748383 10.1073/pnas.1131604100 PMC164504

[55] Dheilly E, Battistello E, Katanayeva N, Sungalee S, Michaux J, Duns G, et al. Cathepsin S regulates antigen processing and T cell activity in Non-Hodgkin lymphoma. Cancer Cell. 2020;37(5):674–89. 32330455 10.1016/j.ccell.2020.03.016

[56] Micevic G, Daniels A, Flem-Karlsen K, Park K, Talty R, McGeary M, et al. IL-7R licenses a population of epigenetically poised memory CD8+ T cells with superior antitumor efficacy that are critical for melanoma memory. Proc Natl Acad Sci U S A. 2023;120(30): e2304319120. 37459511 10.1073/pnas.2304319120 PMC10372654

[57] Frumento G, Verma K, Croft W, White A, Zuo J, Nagy Z, et al. Homeostatic cytokines drive epigenetic reprogramming of activated T cells into a “naive-memory" phenotype. IScience. 2020;23(4): 100989. 32240954 10.1016/j.isci.2020.100989 PMC7115140

[58] Melchionda F, Fry TJ, Milliron MJ, McKirdy MA, Tagaya Y, Mackall CL. Adjuvant IL-7 or IL-15 overcomes immunodominance and improves survival of the CD8+ memory cell pool. J Clin Invest. 2005;115(5):1177–87. 15841203 10.1172/JCI23134 PMC1074679

[59] Nanjappa SG, Walent JH, Morre M, Suresh M. Effects of IL-7 on memory CD8 T cell homeostasis are influenced by the timing of therapy in mice. J Clin Invest. 2008;118(3):1027–39. 18246202 10.1172/JCI32020 PMC2214844

[60] Merchant MS, Bernstein D, Amoako M, Baird K, Fleisher TA, Morre M, et al. Adjuvant immunotherapy to improve outcome in high-risk pediatric sarcomas. Clin Cancer Res. 2016;22(13):3182–91. 26823601 10.1158/1078-0432.CCR-15-2550 PMC7831150

[61] Buggert M, Price DA, Mackay LK, Betts MR. Human circulating and tissue-resident memory CD8 T cells. Nat Immunol. 2023;24:1076. 37349380 10.1038/s41590-023-01538-6

[62] Han J, Khatwani N, Searles TG, Turk MJ, Angeles CV. Memory CD8+ T cell responses to cancer. Semin Immunol. 2020;49: 101435. 33272898 10.1016/j.smim.2020.101435 PMC7738415

[63] Pellegrini M, Calzascia T, Elford AR, Shahinian A, Lin AE, Dissanayake D, et al. Adjuvant IL-7 antagonizes multiple cellular and molecular inhibitory networks to enhance immunotherapies. Nat Med. 2009;15(5):528–36. 19396174 10.1038/nm.1953

[64] Stickdorn J, Nuhn L. Reactive-ester derived polymer nanogels for cancer immunotherapy. Eur Polym J. 2020;124: 109481.

[65] Petruzzella A, Bruand M, Santamaria-Martinez A, Katanayeva N, Reymond L, Wehrle S, et al. Antibody-peptide conjugates deliver covalent inhibitors blocking oncogenic cathepsins. Nat Chem Biol. 2024;20(9):1188–98. 38811854 10.1038/s41589-024-01627-z

