# Supplementary material for: Unraveling Cathepsin S regulation in interleukin-7-mediated anti-tumor immunity reveals its targeting potential against oral cancer
Source: J Biomed Sci. 2025 Jul 24;32:69. doi: 10.1186/s12929-025-01154-6 (PMC12288273; doi:10.1186/s12929-025-01154-6)
Supplement: Supplementary file 1 [file 12929_2025_1154_MOESM1_ESM.docx]

**Fig. S1. Inhibiting CTSS suppresses OC tumor growth *in vivo.*** (A) The expression of CTSS in shControl and sh*ctss* stable-expressed NHRI-HN1 cells. (B) The expression level of cytokines (GM-CSF, IL-1α, IL-1β, IL-2, IL-4, IL-5, IL-6, IL12, IL-13, LIX, IL-17α, KC, MIP2, IFN-γ, TNF-α) is plotted in the dot graph by each sample (N=16 in each group). (C) Mice were implanted with NHRI-HN1 tumors carrying either sh*Ctss* or shControl and were grouped by treatment with αIL-7 or IgG antibodies (N=10 for each group). Tumors were extracted and imaged at the end of the study. The weight of extracted tumors is plotted in the dot-bar graph.

**Fig. S2. The IL-7 level of αIL-7 treatment in the CTSS-knockdown OC synergetic mice model.** The representative microscopy photos of the IHC staining for IL-7 on the tumor slide from all mice.

**Fig. S3. Gating strategy of FACS.** The FACS gating strategy in this study.

**Fig. S4. CTSS remodels the T cell subsets*.*** The dot graph shows the percentage of CD3^+^ T cells in CD45 cells; CD4^+^ T cells, tissue-resident memory CD4 cells, and effector memory CD4^+^ cells in CD4^+^ T cells; Granzyme B^+^, PD-1^+^, CD8^+^ Treg cells, effector CD8^+^ cells, central memory CD8^+^ cells, effector memory CD8^+^ cells, and tissue-resident memory CD8^+^ cells in CD8^+^ cells from the tumor tissue in each group. Each dot represented the result of one mouse.

**Fig. S5. CTSS inhibits IL-7 secretion in OC cell lines and oral keratinocyte cells.** (A & B) The mRNA expression level of *CTSS* in the *CTSS* (A) -silenced or (B) -overexpressed (O/E) OEC-M1, SAS, and TW2.6 cells was analyzed by the real-time PCR after 48-hour transfection. (C) The protein expression level of CTSS in OEC-M1, SAS, TW2.6, and DOK cells. (D) The exocytotic levels of IL-7 were analyzed by the ELISA for *CTSS*-silenced (si*CTSS*) or *CTSS*-overexpressed (O/E *CTSS*) DOK cells. Each dot represented one individual experiment. (E) The expression of CTSB and CTSL in the *CTSB*-silenced (si*CTSB*) or *CTSL*-silenced (si*CTSL*) OEC-M1, SAS, and TW2.6 cells. (F) The ELISA analyzed the exocytotic levels of IL-7 for *CTSB*-silenced (si*CTSB*) or *CTSL*- silenced (si*CTSL*) OEC-M1, SAS, and TW2.6 cells. Each dot represents one individual experiment.

**Fig. S6. CTSS inhibits IL-7 secretion through mediating IL-7R in OC cell lines.** (A) Representative images of the confocal microscopy for IL-7-GFP transfected OEC-M1 cells. IL-7R and V-SNARE were labeled after 48-hour transfection. (B) The dot graph plots correlation between the IHC staining score of CTSS and IL-7R in the HNC tissue array. (C)The ELISA analyzed the exocytotic levels of IL-7 in the *CTSS*-silenced and/or *IL-7R*-silenced SAS and TW2.6 cells. si*IL-7R* is from the Santa Cruz Biotechnology. Each dot in the graphs represents one individual experiment. (D) The ELISA analyzed the exocytotic levels of IL-7 in the *CTSS*-silenced and/or *IL-7R*-silenced OEC-M1, SAS, and TW2.6 cells. si*IL-7R* is from the Dharmmacon. Each dot in the graphs represents one individual experiment. (E&F) The protein expression level of IL-7R was analyzed in *CTSS*-silenced or -overexpressed SAS and TW2.6 cells by the Western blot analysis after 48-hour transfection. (G) The protein-protein interaction was studied by IP for CTSS in SAS and TW2.6 cells followed by IB for IL-7R. Pretreatment with si*CTSS* was done before lysate collection for the IP-IB study.

**Fig. S7. CTSS targets IL-7R on the intracellular domain of IL-7R in OC cells.** (A) The protein-protein interaction of IL-7R or IL-7R1-264 was studied by IP for myc-tag in myc-tagged IL-7R/IL-71-264-transfected SAS and TW2.6 cells followed by IB for CTSS. (B) The ELISA analyzed the exocytotic levels of IL-7 in the *CTSS*-overexpressed SAS and TW2.6 cells. with co-transfection of IL-7R/IL-7R1−264 DNA plasmids. Each dot in the graphs represents one individual experiment. (C) SAS and TW2.6 cells were transfected with myc-tagged *IL-7R, IL-7R1−414*, *IL-7R1−364, IL-7R1−314, IL-7R1−264* plasmid. Myc-tag was pulled down by IP. The expression of CTSS was detected by IB. (D) The ELISA-detected IL-7 levels in the medium of *CTSS*-overexpressed SAS and TW2.6 cells that were co-transfected with myc-tagged *IL-7R, IL-7R 427AA, IL-7R 433AA, IL-7R 445AA, IL-7R 448AA* plasmid are shown in the dot-blot graph.

**Fig. S8. IL-7 vesicles are colocalized with CTSS in early endosome and lysosome** (A) Representative image of confocal microscopy and the fluorescence intensity profiles for the IL-7-GFP-transfected OEC-M1 cells with V-SNARE, CTSS, and Rab5 labeled. (B) Representative image of confocal microscopy and the fluorescence intensity profiles for the IL-7-GFP-transfected OEC-M1 cells with V-SNARE and LAMP1 labeled.

**Fig. S9. Inhibiting CTSS promotes IL-7 secretion in OC cells.** (A) The ELISA detection of the SAS and TW2.6 medium IL-7 is shown in the bar graph with each dot representing one individual experiment. Cells were treated with sucrose for 72 hours. (B) The CTSS-overexpressed OEC-M1 cells were pretreated with 3 nM BafA1 at 1 hour prior, or were cotreated with siLAMP1. The protein expression level of IL-7R in OEC-M1 was detected by the Western blot analysis after 48-hour transfection. (C & D) The exocytic IL-7 in the medium that was detected by ELISA was shown in the dot-bar graph. The *CTSS*-overexpressed SAS and TW2.6 cells were pretreated with (C) 3 nM BafA1 at 1 hour prior or were co-transfected with (D) si*LAMP1* for 48 hours. (E) The effect of RJW-58 on CTSS activity was measured by the cathepsin S activity assay with the results showed in the bar graph. Each dot represented one individual experiment. (F) The exocytic IL-7 in the RJW-58-treated SAS and TW2.6 cells’ medium that was detected by ELISA was shown in the dot-bar graph. (G&H) The effect of curcumin (4 μM), Z-phe-Tyr-CHO (50 μM), and E-64 (50 μM) to (G) CTSS activity and (H) the exocytotic IL-7 was measured by the cathepsin S activity assay and the ELISA, respectively, with the resulted showed in the bar graph. Each dot represents one individual experiment.

**Fig. S10. RJW-58 inhibited tumor growth by increasing the IL-7 *in vivo***. Mice were subcutaneously inoculated with NHRI-HN1 cells. (A) The calculated tumor volume was shown in the curve lines by treatment (N=8 for each group). Red and blue arrowheads indicate days for RJW-58 and antibody administration, respectively. (B) The curve lines show the body weight by treatment groups. (C&D) All mice were sacrificed on day 18. Tumors were extracted. (C) The tumor weight of each mouse was measured. (D) Tumors are shown in the photo. (E) The representative photos of the IHC staining for CTSS, IL-7, and CD8^+^ T-cells by group are shown. (F-H) FACS analysis for the tumor-infiltrative leukocytes, with the percentage of (F) CD3^+^ T cells in CD45^+^ cells, (G) CD4^+^ T cells in CD3^+^ cells, and (H) CD8^+^ T-cells in CD3^+^ cells, Granzyme B^+^ CD8^+^ T cell in CD8^+^ T cell, and PD-1+ CD8^+^ T cell in CD8^+^ T cell of each mouse show in the dot graph.

**Fig. S11. The anti-cancer effect of αPD-1 in CTSS-knockdown cell *in vitro*.** The tumor volume of the subcutaneously-inoculated NHRI-HN1 cell is plotted. Mice were implanted with tumors carrying either sh*Ctss* or shControl, and were grouped by treatment with αPD-1 or IgG antibodies (N=10 for each group). Arrowhead indicates days for antibody administration. However, there was one in each of the shControl+ αPD-1 and sh*Ctss* + αPD-1 groups that died prematurely and were excluded from the result (day 15).

**Fig. S12. RJW-58 and αPD-1 show anti-tumor effects *in vivo*.** (A) The curve plot shows the body weight of all treatments. (B) Tumors were extracted at the end of the study and imaged. (C&D) The dot graph shows the percentage of CD3^+^ T cells, CD4^+^ T cells, CD4^+^ Treg cells, central memory CD4^+^ T cells, tissue resident memory CD4^+^ T cells, and effector memory CD4^+^ T cells from the (C) NHRI-HN1 or (D) MOC-1 cell-bearing tumor tissue in each group. Each dot represented the result of one mouse.

**Fig. S13. The effect of RJW-58 and αPD-1 combination therapy in remodeling CD8^+^ T-cell subsets *in vivo*.** The dot graph shows the percentage of Granzyme B^+^ CD8^+^ T cells, PD-1^+^CD8^+^ T cells, CD8^+^ Treg cells, naïve CD8^+^ T cells, effector memory CD8^+^ T cells, and tissue resident memory CD8^+^ T cells, and from the (A) NHRI-HN1 or (B) MOC-1 cell-bearing tumor tissue in each group. Each dot represents the result of one mouse.

**Fig. S14. The CD8^+^ T-cell infiltration level of combination RJW-58 and αPD-1 in OC synergetic mice model.** The representative microscopy photos of the IHC staining for CD8^+^ on the tumor slide from all mice are shown.

**Fig. S15. The localization of IL-7 vesicles in OC cells.** Representative images of confocal microscopy for the IL-7-GFP/IL-7R-mRFP-trasnfected OEC-M1 cells with Calnexin, GOLGA5, and E-cadherin labeled after 48-hour transfection.

**Fig. S16. IL-7 interacts with the common gamma chain in the cytoplasm.** IL-7 in OEC-M1 lysate was pulled down by IP. The expression of IL-2 receptor gamma chain (IL-2Rγ) was then detected by Western blotting.


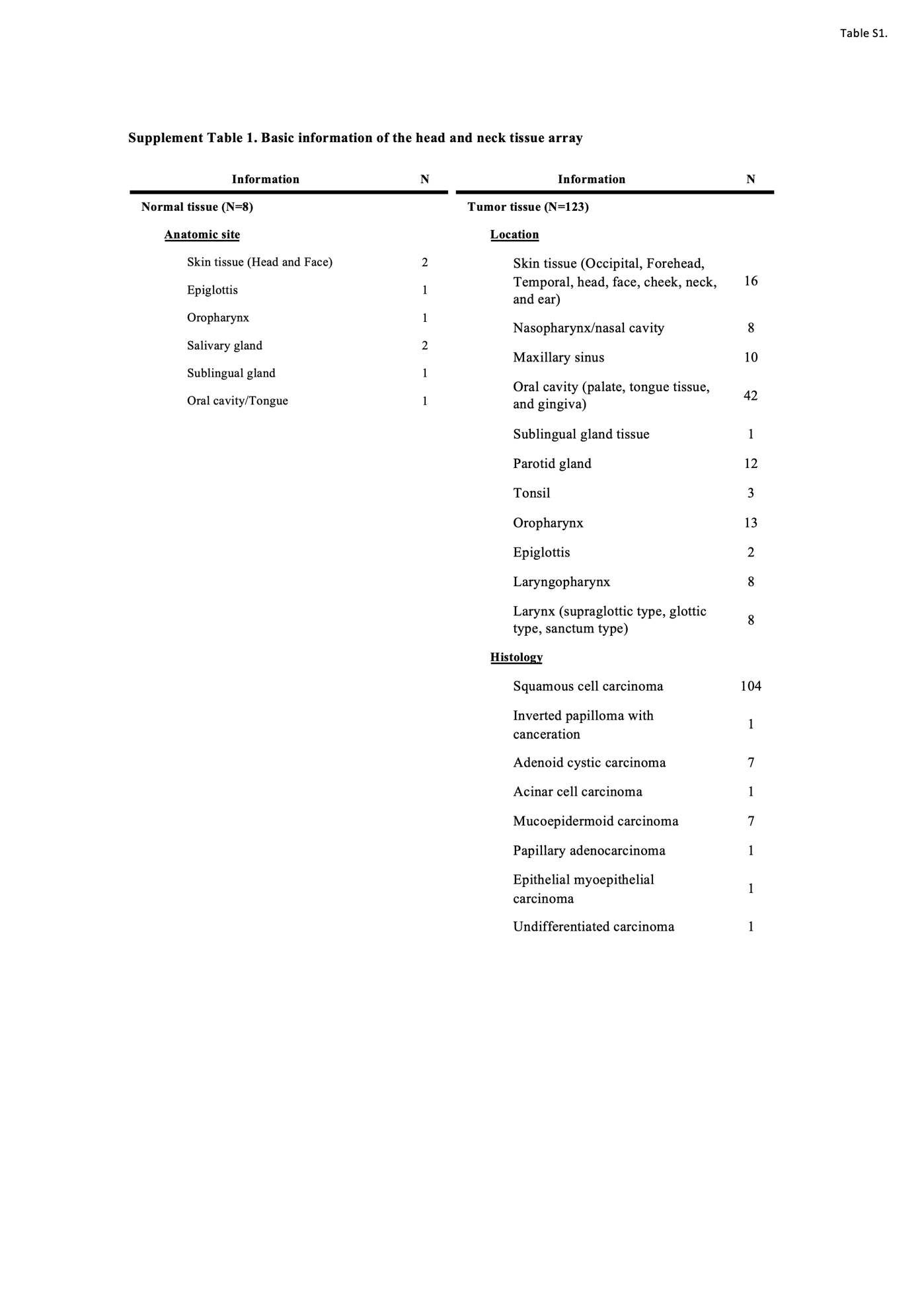


**Table S1. Basic information of the head and neck tissue array.**


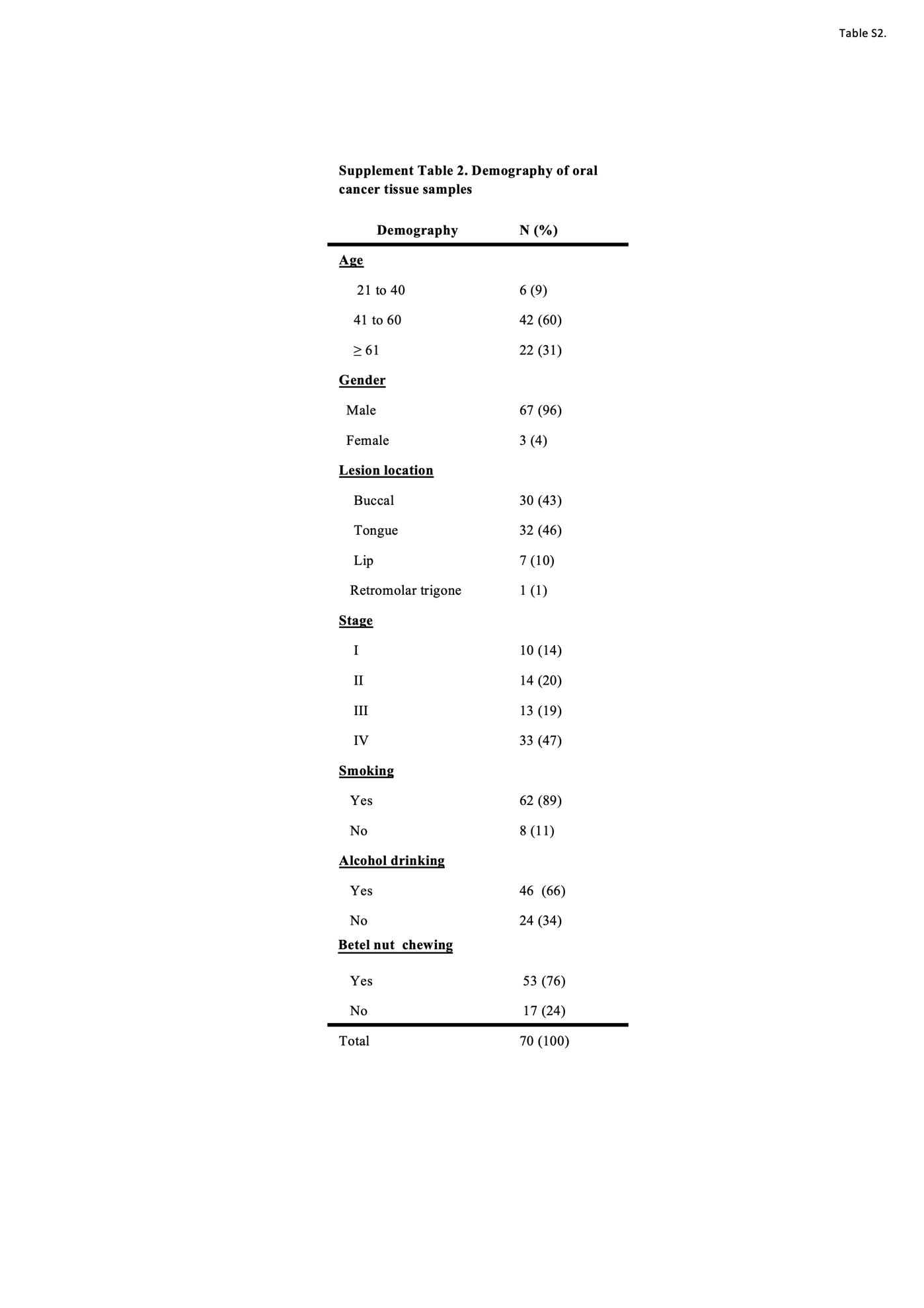


**Table S2. Demography of oral cancer tissue samples.**
